# Genetic changes in a novel breeding population of *Brassica napus* synthesized from hundreds of crosses between *B. rapa* and *B. carinata*

**DOI:** 10.1111/pbi.12791

**Published:** 2017-08-16

**Authors:** Jun Zou, Dandan Hu, Annaliese S. Mason, Xueqi Shen, Xiaohua Wang, Nian Wang, Fabian Grandke, Meng Wang, Shihao Chang, Rod J. Snowdon, Jinling Meng

**Affiliations:** ^1^ National Key Laboratory of Crop Genetic Improvement Huazhong Agricultural University Wuhan China; ^2^ Department of Plant Breeding IFZ Research Centre for Biosystems Land Use and Nutrition Justus Liebig University Giessen Germany; ^3^ College of Horticulture & Forestry Sciences Huazhong Agricultural University Wuhan China

**Keywords:** SNP, resynthesized, interspecific hybridization, genome changes, selective sweep, *Brassica*

## Abstract

Introgression of genomic variation between and within related crop species is a significant evolutionary approach for population differentiation, genome reorganization and trait improvement. Using the Illumina Infinium *Brassica* 60K SNP array, we investigated genomic changes in a panel of advanced generation new‐type *Brassica napus* breeding lines developed from hundreds of interspecific crosses between 122 *Brassica rapa* and 74 *Brassica carinata* accessions, and compared them with representative accessions of their three parental species. The new‐type *B. napus* population presented rich genetic diversity and abundant novel genomic alterations, consisting of introgressions from *B. rapa* and *B. carinata*, novel allelic combinations, reconstructed linkage disequilibrium patterns and haplotype blocks, and frequent deletions and duplications (nonrandomly distributed), particularly in the C subgenome. After a much shorter, but very intensive, selection history compared to traditional *B. napus*, a total of 15 genomic regions with strong selective sweeps and 112 genomic regions with putative signals of selective sweeps were identified. Some of these regions were associated with important agronomic traits that were selected for during the breeding process, while others were potentially associated with restoration of genome stability and fertility after interspecific hybridization. Our results demonstrate how a novel method for population‐based crop genetic improvement can lead to rapid adaptation, restoration of genome stability and positive responses to artificial selection.

## Introduction

Interspecific hybridization is a significant evolutionary process in crop speciation and a frequently used approach for crop genetic improvement (Abbott, 1992; Ellstrand and Schierenbeck, 2000; Hajjar and Hodgkin, 2007; Kuligowska *et al*., 2016; Mallet, 2005; Rieseberg and Carney, 1998). It is estimated that 25% of all plant species have undergone the process of interspecific hybridization in nature, and this process is thought to broaden genetic diversity and confer evolutionary advantages (Mallet, 2005). During interspecific hybridization, rapid genomic alterations, such as transposon activation, transcriptional and epigenetic modification, and genomic structural variation which can result in potentially beneficial phenotypic variation, were attractive for crop germplasm innovation and thus frequently explored (Adams *et al*., 2004; Gaeta *et al*., 2007; Pires *et al*., 2004; Rieseberg and Ellstrand, 1993; Shaked *et al*., 2001; Shen *et al*., 2014; Singh *et al*., 2005; Soltis *et al*., 2016; Xiong *et al*., 2011; Zou *et al*., 2011). However, interspecific crosses often have strong hybridization barriers and require time‐consuming selection following rapid segregation (Kuligowska *et al*., 2016; Lowry *et al*., 2008; Rieseberg and Carney, 1998). Often, offspring in early generations have poor fertility and genome stability (Cifuentes *et al*., 2010; Mason and Batley, 2015). Additionally, favourable traits introgressed from other species into crops are often tightly linked with undesirable traits, requiring several rounds of recombination and a lot of effort to break these associations (Lewis *et al*., 2007). Therefore, understanding the factors and mechanisms impacting recombination frequency, genome stability, cross‐compatibility and selective sweeps would allow for better manipulation of interspecific hybridization to improve traits such as fertility, resistance and yield (Bomblies, 2010).

The *Brassica ‘*U's Triangle’ species (U, 1935) offer an ideal model system for utilizing and understanding the process of interspecific hybridization, particularly in crops (Mason and Snowdon, 2016). The *Brassica* ‘U's Triangle’ consists of three diploid species: *B. rapa* (A^r^A^r^), *B. nigra* (B^n^B^n^) and *B. oleracea* (C^o^C^o^), and three derived tetraploid species: *B. juncea* (A^j^A^j^B^j^B^j^), *B. napus* (A^n^A^n^C^n^C^n^) and *B. carinata* (B^c^B^c^C^c^C^c^). These species readily hybridize (FitzJohn *et al*., 2007), and allopolyploid species benefit from genomic plasticity conferred by frequent homoeologous sequence exchanges between their two subgenomes (Chalhoub *et al*., 2014; Liu *et al*., 2014; Yang *et al*., 2016). Abundant genomic variation within and between species also offers strong potential for crop improvement via interspecific hybridization (Zou *et al*., 2010). On the one hand, rich subgenomic variation exists within each of the *Brassica* A, B and C genomes as a result of speciation, domestication and geographic differentiation (Chalhoub *et al*., 2014; Liu *et al*., 2014; Wang *et al*., 2011). On the other hand, rich post‐Neolithic variation exists between subgenomes as a result of hybridization, polyploidization, domestication and artificial selection for different human uses, such as between the A^r^/A^j^/A^n^ subgenomes from *B. rapa*,* B. juncea* and *B. napus* (Yang *et al*., 2016; Zou *et al*., 2016). Furthermore, early‐modern variation exists within species (diverse lines), resulting from processes such as artificial selection and genetic improvement. Examples include cultivated variation within each of *B. napus*,* B. rapa* and *B. oleracea* (Cheng *et al*., 2016; Liu *et al*., 2016; Qian *et al*., 2014; Wang *et al*., 2014). Therefore, exploring subgenomic variation in *Brassica* is potentially very useful in theoretical and applied research.

The predominant oilseed *Brassica* species currently grown worldwide, *B. napus*, is a relatively young species, formed no more than a few thousand years ago (Chalhoub *et al*., 2014). *Brassica napus* has also only undergone ~400 years of domestication and cultivation, resulting in a narrow genetic base in this crop (Chalhoub *et al*., 2014). This is also evident in the genome as large blocks of linkage disequilibrium, particularly in the C subgenome (Qian *et al*., 2014; Wang *et al*., 2014), which may be related to strong selection pressures and linkage drag in the breeding of ‘double‐low’ seed quality of *B. napus*. Therefore, introducing novel genetic diversity into the A and especially C subgenome is highly desirable to further broaden the genetic base of *B. napus*. To this end, major effort has been put towards exploring and utilizing subgenomic variation within each of the *Brassica* A, B and C subgenomes and between species for *B. napus* crop improvement. Both targeted gene transfer and whole‐genome introgressions into *B. napus* have been carried out via interspecific crosses, significantly broadening the genetic base of *B. napus* and promoting trait improvement and hybrid heterosis (Becker *et al*., 1995; Chatterjee *et al*., 2015; Chen *et al*., 2010; Fu *et al*., 2012; Li *et al*., 2014b; Rahman, 2001; Schranz and Osborn, 2000).

With the aim of exploiting subgenomic variation between genomes, between species and within species, we created a ‘new‐type’ *B. napus* by replacing the A^n^C^n^ genomes with A^r^ and C^c^ subgenomes from multiple accessions of *B. rapa* and *B. carinata*, respectively, in our previous studies (Xiao *et al*., 2010). Subsequently, an advanced germplasm pool of new‐type *B. napus* was established via six to seven additional rounds of recombination and selection (unpublished). In addition to the introduction of within‐species genetic diversity in *B. rapa* and *B. carinata* into new‐type *B. napus*, we hypothesized that an additional level of genomic variation, novel genomic alterations induced by the hybridization event, would be generated genomewide. In this advanced germplasm pool of new‐type *B. napus*, our objectives were (i) to analyse the genetic variation within the new‐type *B. napus* population; (ii) to characterize breakage, reconstruction and fixation of LD and haplotype blocks in this population; (iii) to investigate the presence of novel genetic variation across the genome; and (iv) to find signals of agricultural selective pressure and assess the role they played in the establishment of a new ‘species type’. These analyses help reveal genome evolutionary processes after massive exotic genome introgressions into an established species.

## Results

### Genetic diversity in the new‐type *B. napus* population: substantial population differentiation compared with the parental species

**Figure 1:**
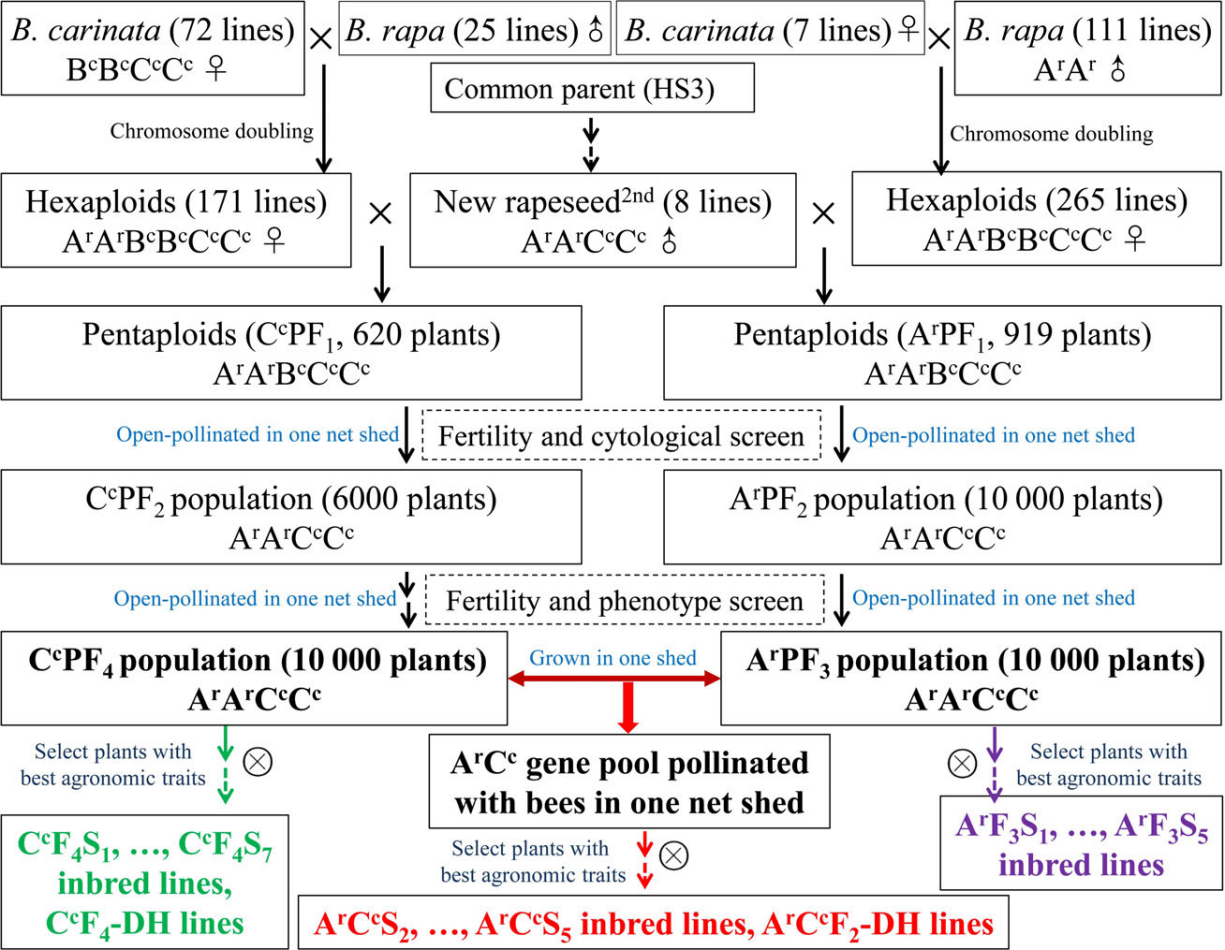
Construction of the novel inbred breeding population of *Brassica napus* diversified in both the A and C genomes. A total of 74 accessions of *B. carinata* and 122 accessions of *B. rapa* were involved in the gene pool, and five accessions of *B. carinata* and 13 accession of *B. rapa* were used in generation of both the C^c^ population and the A^r^ population.

A total of 130 inbred lines of the new‐type *B. napus* (BnN) were bred from hundreds of interspecific crosses involving 74 accessions of *B. carinata* and 122 accessions of *B. rapa* (Figure 1; Table S1). Together with 15 *B. rapa* accessions (Br), 14 *B. carinata* accessions (Bc) and 130 traditional *B. napus* accessions (BnT), the BnN lines (Table S2) were genotyped using *Brassica* 60K Illumina Infinium SNP arrays. After marker filtering, a total of 33 541, 13 958 and 3990 polymorphic SNPs were scored in the populations of BnN, Br and Bc, respectively (Table S3), and a total of 37 473 polymorphic SNPs were scored in the whole four populations and used to evaluate the population differentiation. The average PIC (0.29) and gene diversity (0.36) within the BnN population were the same for both the A and C genomes (Table S3). The mean PIC, gene diversity and genetic distance (average value of 0.27) within the BnN population were comparable to that presented within the BnT population (Tables S3 and 1). These results indicated comparable genetic diversity in the new‐type *B. napus* population evaluated with the 60K‐SNP array compared with that of the traditional *B. napus* population.

**Table 1:** Genetic differentiation within and between the new‐type *Brassica napus* and its parental species using a total of 37 473 polymorphic SNP markers

Population	BnN	BnT	Br	Bc
BnN	0.268^a^ (0.001–0.401^b^)	0.120	0.265	0.317
BnT	0.194	0.289 (0.002–0.483)	0.305	0.364
Br	0.182	0.170	0.193 (0.138–0.224)	0.613
Bc	0.290	0.280	0.239	0.036 (0.024–0.047)

Genetic distance estimates appear below the diagonal and pairwise *F*_ST_ above the diagonal (with grey backdrop).

a The average genetic distance between the individuals within the population.

b Numbers in brackets are the range of the genetic distance between the individuals within the population.

**Figure 2:**
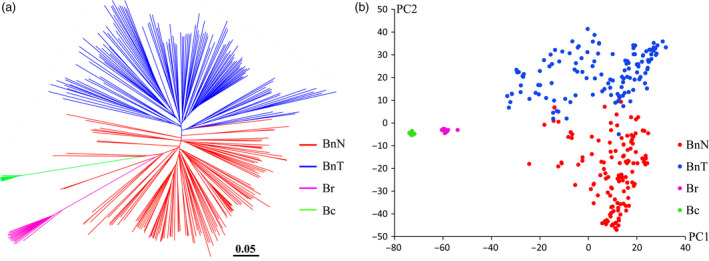
Genetic clustering of the new‐type *Brassica napus* lines and comparison with the parental species using a total of 37 473 polymorphic SNP markers. (a) Genetic clustering of the investigated *Brassica* accessions from the BnN (new‐type *B. napus*, 130 accessions), BnT (traditional *B. napus*, 130 accessions), Br (*B. rapa*, 15 accessions) and Bc (*B. carinata*, 14 accessions) populations based on the genetic distance. (b) PCA of the BnN, BnT, Br and Bc populations.

Four apparent genetic clusters were observed for each of the BnN, BnT, Br and Bc populations (Figure 2a). The first and second principal components explained more than 90% of the total genetic difference among the four populations (Figure 2b). There was substantial genetic distance and *F*_ST_ between the new‐type *B. napus* and the other three subpopulations (*B. rapa*,* B. carinata* and traditional *B. napus*), while the new‐type *B. napus* showed less genetic distance to traditional *B. napus* than to *B. rapa* and *B. carinata* (Table 1). The *F*_ST_ between BnN and BnT was twice as small as that between species (*B. rapa* and *B. carinata*). Substantial population differentiation was observed between the BnN and BnT populations, but the population differentiation within species was smaller than that between species, such as that between the BnN and Br/Bc populations (Table 1).

### Breakage and reconstruction of linkage disequilibrium and haplotypes in the new‐type *B. napus* population

**Figure 3:**
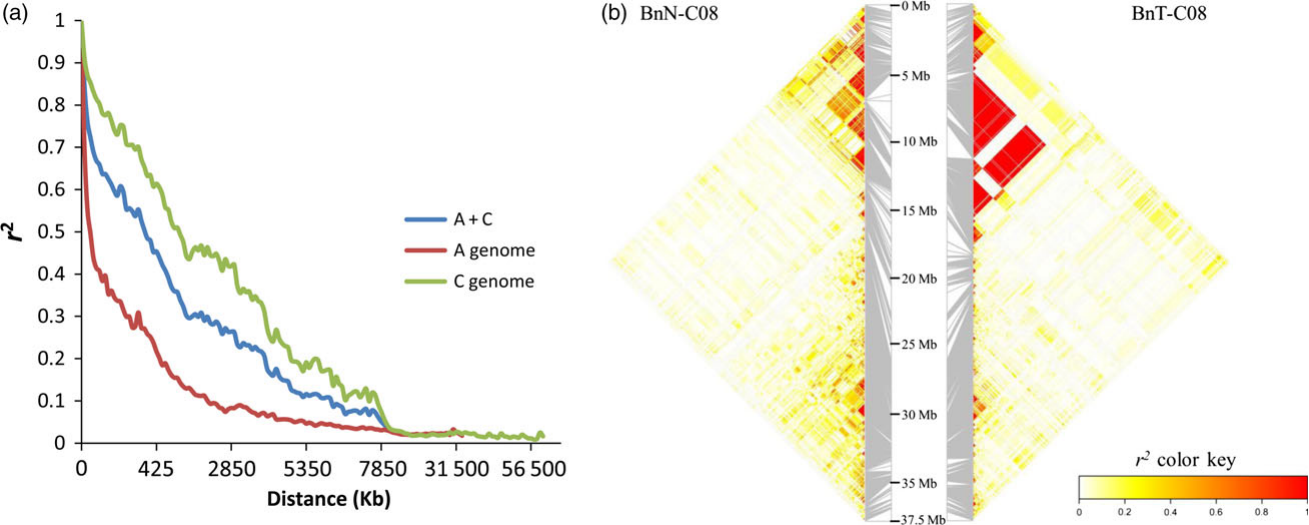
The pattern of LD decay in the new‐type *Brassica napus* population. (a) LD decay distance in the new‐type *B. napus* population. (b) Broken and reconstructed LD patterns on chromosome C08 in the BnN (new‐type *B. napus* population) compared with BnT (traditional *B. napus*). The strong LD blocks in BnT located from 5.47 to 12.25 Mb were reconstructed as several small, weak LD blocks in the BnN population.

The pattern of LD in the new‐type *B. napus* population was analysed using a total of 19 112 SNPs with unique alignment positions to the reference genome, Darmor‐*bzh*, after filtering out the markers with >20% heterozygosity. In general, the LD pattern varied among chromosomes and subgenomes (Table 2). The A genome presented a much more rapid LD decay rate and shorter LD decay distance than the C genome (Figure 3a), and apparent reconstruction of LD was found in the new‐type *B. napus* population on some chromosomes, such as C02, C03 and C08 (Figures 3b and S1). Large LD blocks were consistently located in the *B. napus* centromere regions (Mason *et al*., 2016), particularly in C01, C04 and C07 (Figure S1), with similar results in both the BnN and BnT populations.

**Table 2:** Summary of the linkage disequilibrium and haplotype blocks in the population of new‐type *Brassica napus*

Chromosome	No. of SNPs	Mean *r* ^2^	LD decay to half (Mb)	LD decay to 0.1 (Mb)	Number of haplotype blocks	Average number of haplotypes for each block (max)
A genome						
A01	910	0.06	0.01–0.03	1.5–1.7	132	3.68 (9)
A02	684	0.08	0.01–0.03	1.5–1.7	93	4.14 (9)
A03	1265	0.05	0.01–0.03	1.5–1.7	207	3.73 (10)
A04	868	0.06	0.01–0.03	1.4–1.6	122	3.72 (9)
A05	974	0.05	0–0.02	1.1–1.3	149	3.81 (9)
A06	947	0.06	0.01–0.03	1.7–1.9	140	3.94 (9)
A07	1166	0.06	0–0.02	1.1–1.3	170	4.08 (17)
A08	717	0.11	0.02–0.04	4.7–4.9	97	4.18 (9)
A09	1002	0.08	0.19–0.25	2.4–2.6	126	3.92 (15)
A10	936	0.11	0.02–0.04	4.2–4.4	107	3.66 (10)
Mean	946.90	0.07	0.02–0.05	2.11–2.31	134.3	3.89 (10.60)
Subtotal	9469	–	–	–	1343	–
C genome						
C01	1658	0.27	3.90–4.10	7.9–9.0	78	3.95 (18)
C02	250	0.12	0.20–0.30	7.1–7.3	24	5.13 (11)
C03	1682	0.05	0.11–0.13	2.3–2.5	143	3.91 (13)
C04	2215	0.22	2.90–3.10	5.8–6.0	101	3.56 (10)
C05	603	0.09	0.09–0.11	1.8–2.0	69	3.41 (13)
C06	780	0.07	0.08–0.10	3.1–3.3	74	3.84 (9)
C07	1112	0.11	0.20–0.30	6.2–6.4	113	3.64 (17)
C08	730	0.08	0.03–0.05	2.7–2.9	77	4.32 (10)
C09	613	0.08	0.18–0.20	2.2–2.4	51	3.75 (8)
Mean	1071.44	0.12	0.85–0.93	4.34–4.64	81.11	3.95 (12.11)
Subtotal	9643	–	–	–	730	–
Whole genome						
Mean	1005.89	0.10	0.42–0.47	3.17–3.42	107.71	3.92 (11.36)
Total	19 112	–	–	–	2073	–

A total of 2073 conserved haplotype blocks were detected in the new‐type *B. napus* population, spanning 248 Mb or 38.7% of the assembled reference genome of *B. napus* (Figure S2). More haplotype blocks were identified in the A genome, but with smaller average size than in the C genome (Table 2 and Figure S2), which is the same trend as that found in traditional *B. napus*. In general, the large haplotype blocks also exhibited strong LD (Figure S1). The haplotype blocks varied between the two populations: approximately two‐thirds of the haplotype blocks >50 kb in size identified in the BnN population were reconfigured, including formation of new blocks and breakage of old blocks relative to the BnT population (Table S4). For example, two haplotype blocks in A01 and C08 that covered the centromere region were detected specifically in the BnN population, while 22.4% of the haplotype blocks identified in the BnT population were not present in the BnN population (Figure S1).

### Introgression and identity by descent (IBD) of original parents in the new‐type *B. napus* population

Multiple accessions of *B. rapa* and *B. carinata* were involved in breeding new‐type *B. napus* (Figure 1). However, the original parental *B. napus*, Huashuang 3 (HS3), was the only *B. napus* parent of the eight‐second‐generation new‐type *B. napus* lines that were crossed to produce the pentaploids at the start of the current breeding programme. Therefore, HS3 was used as the major control to estimate parental introgression and IBD within the new‐type *B. napus* population. By calculating the average allele frequency for each polymorphic marker within the BnN population that originated from HS3 or from the parental *B. rapa* and *B. carinata* accessions we genotyped, it was estimated that approximately 88.4% of the genome on average was replaced with novel introgressions that were different from the parental *B. napus* HS3, and very few IBD originating from HS3 in 12 lines (about 9.2%). Approximately, the whole genome of new‐type *B. napus* was reconstructed, with little remaining from the original *B. napus* parent HS3 (Figure S3, Table S5).

### Putative deletions and duplications in the new‐type *B. napus* population

**Figure 4:**
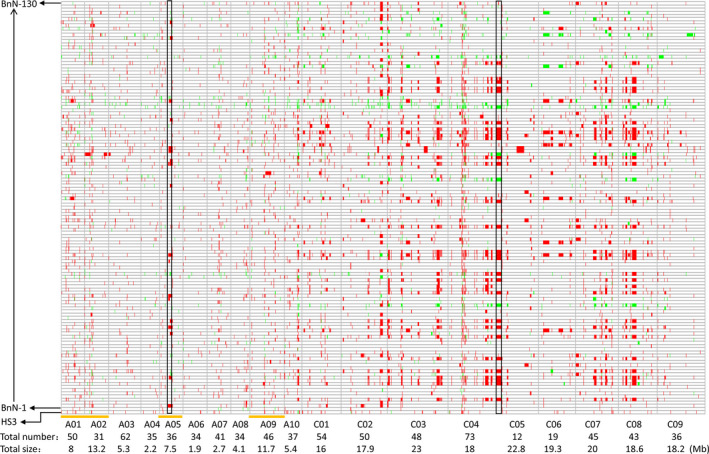
Putative deletions and duplications detected within the population of new‐type *Brassica napus* and HS3. The vertical coordinates represent 130 lines of new‐type *B. napus* and their *B. napus* parent, HS3, and the horizontal axis shows 19 chromosomes. Red and green colours represent deletions and duplications, respectively, across the genome of new‐type *B. napus*. Black borders indicate two big deletion blocks in A05 and C05, respectively. Orange lines shows chromosomes A01, A02, A05 and A09 that had larger deletions/duplication blocks than the other A‐genome chromosomes. The total number and size of the deletion/duplication events of each chromosome are shown underneath each chromosome label.

It was expected that a high number of missing values for considerable markers would be observed in the BnN population, resulting from either exotic introgressions or novel induced variations (such as deletion–duplication events). As expected, we found that 6085 markers presented more than 20% of missing values, even to 100%, across certain regions and especially in the C genome in the BnN population. Thus, using the previously established marker filtering parameters (we initially excluded all markers with more than 20% missing values to estimate population diversity and differentiation compared with the BnT population) would lose many markers involved in novel variation induced in the BnN population, such as deletion events (Table S3). Therefore, we revisited AC markers with unique genomic positions to include those with a high rate of missing values. Finally, a total of 27 506 markers were selected by GSRC for genome structural rearrangement analysis of the BnN population, and its original parental *B. napus*, HS3, as a control. Of these, 39.8% (10 945) were found to be involved in deletion and duplication events in at least one individual of the BnN population, of which a predominance (9935) of the markers were involved with deletions. For the markers with deletion signals, 0.57% of the markers located in the A genome were absent in *B. rapa* and 2.55% of the markers located in the C genome were absent in *B. carinata*. Of the 10 945 total markers, 38.3% and 61.3% were located in the A and C genomes, respectively, which were assigned to a total of 406 and 380 genomic regions covering 26.0% and 42.8% of the A and C genomes, respectively (Figure 4). The average size of duplication/deletion events in the C genome was three times that of duplication/deletion events observed in the A genome. Additionally, chromosomes A01, A02, A05 and A09 had larger deletions and duplication blocks than the other six A‐genome chromosomes, while there was not obvious variation between different C‐genome chromosomes. However, in HS3, a very few (65) deletion/duplication events covering 6.2 Mb were detected, 35 of which (covering 1.8 Mb) overlapped with those detected in the BnN population (Figure 4).

In the putative regions with deletion/duplication events, a total of 453 regions (202 regions in the A genome and 251 regions in the C genome) were large blocks above 50 kb, averaging 538 kb in size and with some regions appearing in up to 30% of the 130 lines (Table S6). For example, a deletion identified in A05 covering 4.8 Mb from physical position 9 606 923 to 14 487 472 bp was present in 3% of lines and a deletion identified in C05 covered 6.8 Mb from physical position 44 364 to 6 852 090 bp and was found in 20.8% of lines (Figure 4). Deletions were identified more often than duplications for the same regions within the population. For example, 30 lines had 4.21 Mb deletion events in C08 from physical position 15 465 176 to 19 679 933 bp, while only four lines showed duplication events in the same region. In general, the duplication/deletion events were frequently detected in noncentromeric regions. However, a few putative deletion/duplications also occurred in the centromeric regions. For instance, a 7.4‐Mb deletion/duplication region detected in C06 from physical position 4 986 118 to 12 387 447 bp was across the centromere region (8.0–8.4 Mb).

### Genomewide selection signals in the new‐type *B. napus* population

A sliding‐window approach (100‐kb windows sliding in 10‐kb steps) was applied to quantify the polymorphism levels (θ_π_), genetic differentiation (*F*_ST_) and selection statistics (Tajima's *D*) within the new‐type *B. napus* lines and between the new‐type *B. napus* and traditional *B. napus* lines. Whole‐genome coverage was provided by all markers over 28 295 windows. In the new‐type *B. napus* population, Tajima's *D* values for 2424 windows was negative, whereas only 20 windows were negative in traditional *B. napus*. The average Tajima's *D* value of the new‐type *B. napus* (0.96) was much lower than that of the traditional *B. napus* (1.74), which suggests genetic bottlenecks in traditional *B. napus*, consistent with previous reports.

**Figure 5:**
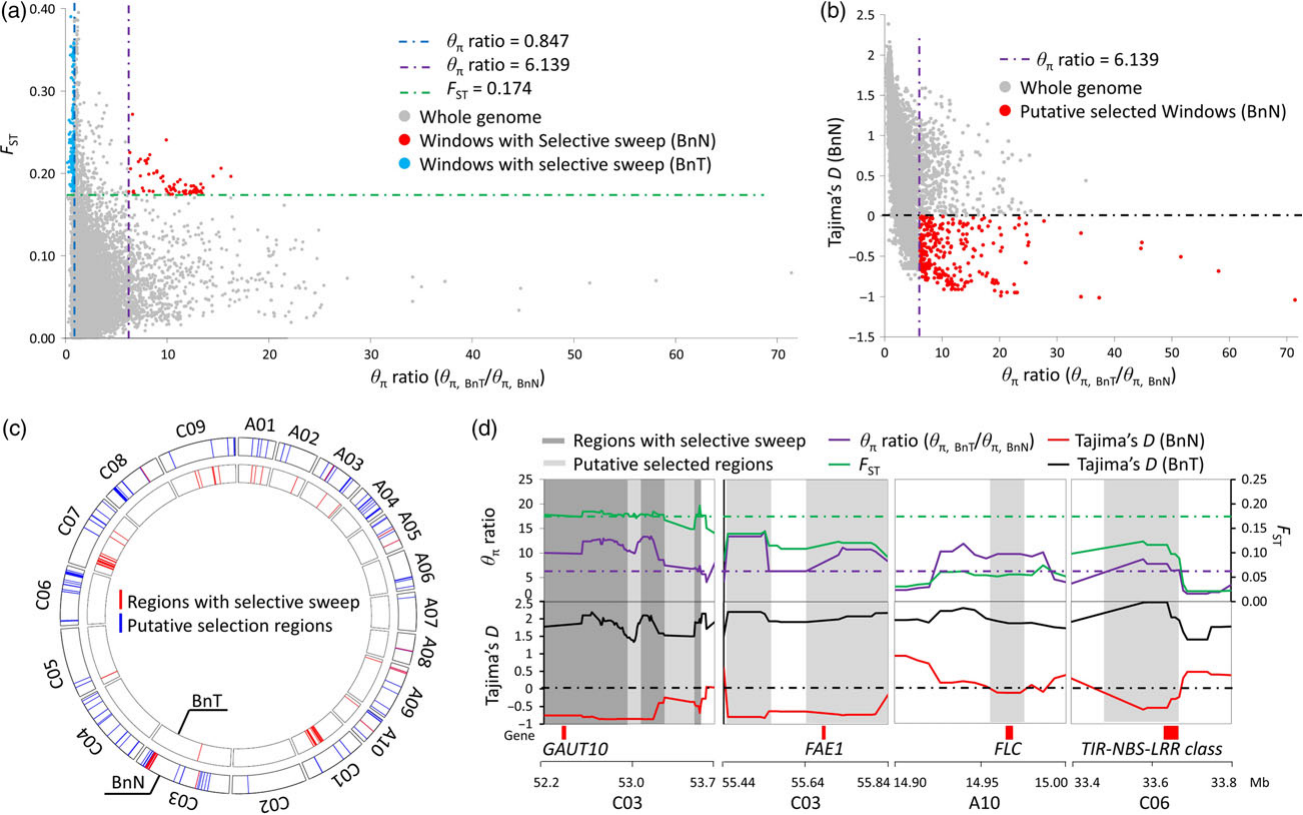
Genomic regions with selective signals in new‐type *Brassica napus* (BnN). (a) The distribution of θ_π_ ratios (θ_π, BnT_/θ_π, BnN_) and *F*_ST_ values, calculated in 100‐kb windows sliding in 10‐kb steps. Dots located to the right of the purple dashed lines (the 5% rightmost tail of the θ_π_ ratios distribution) and above the green dashed line (the 5% rightmost tail of the *F*_ST_ values distribution, where *F*_ST_ is 0.174) were identified as regions with selective sweeps in the BnN population. Blue dots located to the left of the blue dashed lines (the 5% leftmost tail of the θ_π_ ratios distribution) and above the green dashed line (the 5% rightmost tail of the *F*_ST_ values distribution, where *F*_ST_ is 0.174) were identified as regions with selective sweeps in the BnT population. (b) The distribution of selection statistics (Tajima's *D*) and θ_π_ ratios (θ_π, BnT_/θ_π, BnN_) of the new‐type *B. napus*. Data points located to the right of the purple dashed lines (the 5% rightmost tail of the θ_π_ ratio distribution) and below the black dashed line (Tajima's *D* value is 0) were defined as putative selected regions in the BnN population (red points). (c) The distribution of genomic regions with selective signals. The inner and external circles show the BnN population and the BnT population, respectively. Red bars and blue bars represent the regions with selective sweeps and putative selection regions, respectively. (d) Examples of genes within selective sweeps in the new‐type *B. napus* population (BnN). Genomic regions with *F*_ST_ values above the green horizontal dashed line (corresponding to a 5% significance level of *F*_ST_ values) and θ_π_ ratios above the purple horizontal dashed line (a 5% significance level of the θ_π_ ratio) were classed as selective sweep regions in BnN population (deep gray regions). Genomic regions with θ_π_ ratios above the purple horizontal dashed line and negative Tajima's *D* values were classed as putative selected regions for BnN population (gray regions). Genome annotations are shown at the bottom (red bar, genes).

A total of 15 regions (corresponding to 134 windows) with strong selective sweeps were identified in the new‐type *B. napus* population and distributed on seven chromosomes (A03, A05, A08, A09, A10, C03 and C08) covering 2.74 Mb (Table S7, Figure 5a,c). Furthermore, 112 additional putative selected regions were detected across all 19 chromosomes, covering 16.34 Mb (Table S7, Figure 5b,c). In general, more and larger selected regions were detected in the C genome (75 regions covering 12.48 Mb) than in the A genome (52 regions covering 6.60 Mb) (Table S7). For traditional *B. napus*, a total of 46 regions (corresponding to 297 windows) with strong selective sweeps were identified and distributed on ten chromosomes (A01, A02, A03, A09, A10, C01, C03, C05, C07 and C09) covering 7.26 Mb; one putative selected region was detected and involved in the regions with strong selective sweeps (Figure 5a, Table S7). In traditional *B. napus*, more and larger selected regions were detected in the C genome (38 regions covering 6.13 Mb) than in the A genome (eight regions covering 1.13 Mb), a similar trend as in new‐type *B. napus*, but the selected regions were different compared to those of new‐type *B. napus*.

In the selected regions of new‐type *B. napus*, candidate genes and quantitative trait loci (QTL) associated with seed quality traits and important agronomic traits in *B. napus* were observed. For instance, *BnaC.FAE1* (chromosome C03) is known to be associated with erucic acid content (Korber *et al*., 2016), *GAUT10* (chromosome C03) is associated with neutral detergent fibre concentration in seed (negatively correlated with oil concentration) (Li *et al*., 2014a) and *FLC* (chromosome A10) and *CO* (chromosome C09) were known to control flowering time. Nine genes on chromosome C06 were found to code for disease resistance proteins (TIR‐NBS‐LRR class) (Figure 5c), and QTL *qDT50* (A05) and *qDT52* (A05) for seed development rate (Luo *et al*., 2017) were found within or close to the selected regions. Of these regions (15 + 112), 39.4% (6.3 Mb) were involved with large haplotype blocks with strong LD and 37% (6.1 Mb) were involved with deletion/duplication events. For example, the selected regions containing the *FAE1* gene on chromosome C03 were located in a region showing strong LD with a large haplotype block; however, in this region, frequent duplication/deletion events also occurred (deletions observed in 31 lines, duplications observed in five lines). The *FAE1* gene was associated with erucic acid content, and this trait was extensively screened and selected for during the development of the new‐type *B. napus* population. The *B. rapa* parent also showed a deletion in this region. Even though we could not judge whether this results from a specific exotic introgression from *B. rapa* or is an actual deletion resulting from homoeologous exchange, this was a clear example of structural variation fixed in the new‐type *B. napus* population with strong selective sweep signals.

## Discussion

In this study, we investigated the genetic diversity and uniqueness of a novel breeding population of *B. napus* developed from hundreds of interspecific crosses involving 74 accessions of *B. carinata* and 122 accessions of *B. rapa* (Figure 1) using the Illumina Infinium *Brassica* 60K SNP array. As the Illumina Infinium *Brassica* 60K SNP array was developed based on polymorphisms within *B. napus*, much of the specific polymorphism present in the parental *B. rapa* and *B. carinata* lines would not be detectable. Therefore, the genetic diversity of the new‐type *B. napus* population would likely have been underestimated in terms of introgression of A^r,^ B^c^ and C^c^ polymorphism. Despite this, this ‘new‐type’ *B. napus* population still presented comparable genetic diversity to the internationally representative germplasm set of 130 traditional *B. napus* accessions used in this study (Xu *et al*., 2016), as assessed by the number of polymorphic markers, PIC and genetic distance within the population (Tables S3 and 1). These results indicate that the rich genetic diversity present within the newly resynthesized population is comparable to the gene pool of traditional *B. napus*, the latter of which has undergone decades of breeding efforts. Meanwhile, the new‐type *B. napus* population presented obvious differences to the population of traditional *B. napus,* as well as to *B. rapa* and *B. carinata*, as revealed by *F*_ST_ values, genetic clustering and principle component analysis (Tables 1 and 2, Figure 2).

Compared to the single parental cultivar of *B. napus* involved in the pedigree of new‐type *B. napus*, HS3, nearly the whole genome of individuals in the new‐type *B. napus* has been changed (Figure S3, Table S5). In total, 88.4% of the genome was changed or reconstructed via exotic introgressions from *B. rapa* and *B. carinata* and by novel genetic changes, such as frequent putative deletions and duplications covering 25.95% of the genome, even including some centromeric regions. Thus, we observed obvious changes in LD pattern and haplotype blocks in both the A and C genomes (Figures 3, S1 and S2). The reconstructed LD and haplotype patterns may also have resulted from novel allelic combinations. The C subgenome of *B. napus* in particular has very slow LD decay and limited genetic diversity; as such, its genetic basis needs to be improved (Qian *et al*., 2014; Wang *et al*., 2014). *B. carinata*, one of the founder parent species of new‐type *B. napus* population, shows a very fast LD decay in C genome compared to traditional *B. napus*, as reported by Liu *et al*. (2016) and Zhang *et al*. (2017). This may explain the shorter distance when *r* ^2^ drops to half in the new‐type *B. napus* population. On the other hand, extensive pan‐genomic variation may exist in the presently analyzed populations which may not be visible when using markers aligned to the reference genome Darmor‐*bzh*: a great deal of genomic variation and recombination may be undetectable when using only Darmor‐*bzh* as a reference. This limitation may also impact the estimation of the LD and IBD. In future studies, with the availability of more genomic resources, deep analyses of these populations could be better performed based on pan‐genomic comparisons.

Previous research showed that interspecific hybridization can not only introduce exogenous variation but can also rapidly change the genome via as increased chromosome recombination (Nicolas *et al*., 2012; Pontes *et al*., 2004), loss and/or gain of parental DNA fragments and appearance of novel genetic variation (Song *et al*., 1995). In our study, with intensive recombination and hundreds of interspecific crosses, a great number of novel genomic changes were induced in the C subgenome (Figure 4), including frequent deletion/duplications, altered and shorter LD decay lengths, broken LD blocks and changed haplotype blocks, which together demonstrate the efficacy of our approach in inducing novel genetic variation in the C genome. Regions that still showed strong LD similar to that observed in traditional *B. napus* may indicate areas of limited recombination, either due to the same genomic factors inhibiting recombination in *B. napus*, or due to some form of linkage drag. More recombination may be further introduced to break the LD blocks in the new‐type *B. napus*. As well, changed haplotype or LD blocks in the new‐type *B. napus* population that differ from those in the traditional *B. napus* population may be significant for promoting intersubgenomic heterosis or trait improvement when crosses are made between these two populations.

With respect to the deletion/duplication events detected in the new‐type *B. napus* lines, these were labelled as ‘putative deletion/duplication’ events, especially with regard to the deletions, as these could have resulted from homoeologous exchanges coupled with segregation. These regions could also appear to be ‘missing’ because of poor primer binding due to sequence divergence in these regions, resulting from specific exotic introgression segments from the A^r^ and C^c^ subgenomes, as well as the absence of the alignment to the reference genome due to pan genome variation. A number of markers with deletions (0.55% in the A genome and 28.59% in the C genome) were also found to be missing in *B. rapa* and *B. carinata*, respectively, which may indicate specific exotic introgressions. However, we could not conclusively determine whether these events in the new‐type *B. napus* population were due to inheritance, because not all parental *B. rapa* and *B. carinata* accessions could be genotyped: more in‐depth analysis could be performed after appropriate genotyping of all the parents. In general, novel genomic variation occurred much more frequently in the C genome than in the A genome, and homoeologous exchanges from A to C were more frequent than from C to A, as was previously found in resynthesized and natural *B. napus* (Chalhoub *et al*., 2014; Schmutzer *et al*., 2015). It may also indicate that the subgenomic variation between C^c^ and C^n^ was much more than that between A^r^ and A^n^. As a result, many much more missing segments were detected in the C genome of the new‐type *B. napus*. Certain novel genomic variants, whether due to exotic introgressions or due to induced novel structural variation, may occur preferentially and hence be detected at higher frequencies. On the other hand, the overlap with selected regions in advanced generation lines of the new‐type *B. napus* suggests that fixation of particular novel genetic variants may be under selection, and hence be beneficial for genome stability and rapid adaptation of synthetic *Brassica* lines and interspecific hybrid progeny. Thus, these ‘new‐type’ lines could comprise a good material for understanding the rapid genome evolution of *B. napus*, as well as useful bridge materials for creating new germplasm to bring novel diversity and broken linkage disequilibrium blocks into *B. napus*.

New‐type *B. napus*, which was bred by hundreds of interspecific crosses and intensive selection over several years, has an extremely short breeding history compared to natural *B. napus*. Unsurprisingly, the offspring derived from interspecific crosses in the early generations usually had poor fertility and agronomic traits. After intensive selection and recombination, elite alleles and genotypes were fixed, restoring good fertility and stable genomes in the new‐type *B. napus*. This process would putatively result in strong selective sweeps, indicating associations with important agronomic traits, fertility and genome stability. By analysing the genetic diversity within the new‐type *B. napus* population and comparing it to the traditional *B. napus* population, this study detected 15 genomic regions showing strong signals of selective sweeps, as well as 112 additional regions with weaker (potential) selective sweeps (Table S7). In these regions, we found genes associated with important traits that were targeted during the process of constructing the new‐type *B. napus*, such as low erucic acid, appropriate flowering time for semi‐winter environments, disease resistance and other traits of interest (Figure 5). For example, we performed strong selection on seed quality traits of the new‐type *B. napus*, and most lines were subsequently found to have a very low concentration of erucic acid (Table S2). Regions with selective sweeps may also be associated with fertility and genome stability, but to date, little is known about the underlying genetic factors contributing to these traits in *Brassica* interspecific hybrids.

Differentiation between new‐type *B. napus* and traditional *B. napus* was smaller than that observed between species, suggesting that new‐type *B. napus* lines may be readily utilized as a novel gene pool to cross with the traditional gene pool for heterosis breeding. During the selection of new‐type *B. napu*s, we found several novel agronomic traits of interest. For example, several lines had high seed weight from the primary results: the 1000‐seed weights of lines BnN‐9 and BnN‐22 were 5.23 ± 0.21 and 5.25 ± 0.05, respectively, when grown in Wuhan, China (semi‐winter growth environment) in 2013–2014 (Table S2). Other favourable traits such as high linoleic acid content (28.87%), high linolenic acid content (13.30%) (Table S2), shattering resistance and disease resistance have also been observed in this novel gene pool (unpublished), but need further statistical validation in additional field trials in future studies to confirm and obtain complete data.

In our study, it is likely that we significantly underestimated the degree of novel variation resulting from exotic introgressions and structural variation present in the new‐type *B. napus* lines as a result of genotyping using the Illumina Infinium 60K *Brassica* array. This is because SNPs on this array were designed solely to be polymorphic within traditional *B. napus* accessions (Clarke *et al*., 2016). Thus, much of the subgenomic variation between the A^r^ and C^c^ genomes and the A^n^C^n^ genomes is expected to be underrepresented. This also applies to the subgenomic variation between different parental accessions of the *B. rapa* and *B. carinata*. Additionally, it is not possible to detect either chromosomal inversions or balanced reciprocal translocation events using SNP markers in this population, and these events are likely to have occurred. We also could not assess whether B genome introgression from the *B. carinata* parent due to A/B or B/C homoeologous exchanges had occurred using the array, and the new *Brassica* 90K Illumina Infinium SNP array containing A/B/C genome markers, as well as the availability of a complete *B. carinata* reference genome (Parkin I.A.P., personal communication) would advance the work effectively. Therefore, deeper analyses of the genomic structural variation present in this population using more sophisticated sequencing methods would be desirable. In the future, we hope to build on this understanding of how novel genomic alterations occur with substantial exotic genome introgressions and to assess how deeply these impact agronomic traits, especially seed yield traits, and to explore heterosis breeding using these novel *Brassica* polyploids.

## Materials and methods

### The development of the new‐type *B. napus* lines used in this study

In our previous study, third‐generation lines of new‐type *B. napus* (abbreviated as BnN in this study) were developed from hundreds of interspecific crosses involving 74 accessions of *B. carinata* and 122 accessions of *B. rapa* (Figure 1) (Xiao *et al*., 2010; unpublished data). First, 171 successful hexaploid combinations involving 72 *B. carinata* and 25 *B. rapa* accessions as the original parents were crossed with eight selected lines of second‐generation new‐type *B. napus* (selected for ~75% exotic subgenome introgression and double low seed quality) to obtain pentaploids (AABCC) (Xiao *et al*., 2010). One Chinese *B. napus* cultivar with double‐low seed quality traits, HS3, which was bred with introgressions from *B. rapa*, was involved as an common parent of second‐generation new‐type *B. napus* and was therefore designated the common parent of the third‐generation lines. At the start of the project, all the pentaploid plants were grown in one net shed with open pollination, and seeds were harvested and re‐sown up until the F_4_ generation to encourage full recombination among the lines. In the F_2_ generation, the plants with *B. carinata*/*B. rapa* morphological phenotype and very low fertility (most likely aneuploids) were eliminated, then the chromosome number of remaining F_2_ plants were counted through cytological observation, such that only the plants with 38 chromosomes were selected to yield the F_3_ generation. From the F_3_ generation, only phenotypic selection was performed to select plants with high fertility (particularly seed number), *B. napus* morphological phenotypes, best resistances to field‐based pathogens and lowest glucosinolate and erucic acid content in each generation. This population was designated the new‐type *B. napus* C^c^ population, and the construction and selection of lines in this population was elaborated on Xiao *et al*. (2010). From the F_4_ generation, the plants in the new‐type *B. napus* C^c^ population with the best performance were self‐pollinated by single seed descent for seven generations to generate inbred lines (Figure 1; in green). These lines are subsequently referred to as C^c^F_4_S_7_ inbred lines. The best plants in the F_4_ generation were also selected for rapid purification by microspore culture and are subsequently referred to as C^c^F_4_‐DH lines.

Using the same synthesis strategy as for the C^c^ population (which contained diverse C^c^ genome introgressions from 72 starting *B. carinata* accessions), a total of 265 hexaploid combinations from seven *B. carinata* (five of which were also used for the synthesis of the C^c^ population) and 111 *B. rapa* accessions (13 of which were also used for the synthesis of the C^c^ population) were crossed with the same eight selected lines of second‐generation new‐type *B. napus* to generate pentaploids followed by selection and open pollination in a single net shed to the F_3_ generation. This population was subsequently designated the new‐type *B. napus* A^r^ population (containing diverse A^r^ genome introgressions from 111 *B. rapa* accessions). Selected lines from the F_3_ generation of the A^r^ population were inbred for five generations and are subsequently referred to as A^r^F_3_S_5_ inbred lines (Figure 1; in purple); microspore culture has not yet been carried out on this material.

For the construction of new‐type *B. napus*, our objective was to combine the A^r^ and C^c^ populations to produce a diverse A^r^C^c^ gene pool (Figure 1; in red). Therefore, we grew the C^c^F_4_ population together with the A^r^F_3_ population in one large net shed with two rows for each line and a ratio of 1:1 for plants from each population (unpublished). During the flowering period, bees were put in the net shed to assist in open pollination. Phenotypic screening was also performed for each generation from the seedling stage to harvest. The best lines were selected out of the net shed based on selection for agronomic traits (higher seed number, high seed weight), seed quality (lower glucosinolate content and erucic acid content and higher oil content) and environmental adaptability (suitable flowering time, disease resistance and lodging resistance). From the F_3_ generation, lines selected from the A^r^C^c^ gene pool were self‐pollinated by single seed descent for five generations to generate inbred lines. Those lines are subsequently referred to as A^r^C^c^S_2_–A^r^C^c^S_5_ inbred lines. The best F_2_ generation plants were also selected for rapid purification by microspore culture and are subsequently referred to as A^r^C^c^F_2_‐DH lines.

In this study, a total of 130 new‐type *B. napus* lines (coded BnN‐1 to BnN‐130) were investigated, including 10 C^c^F_4_S_5_ inbred lines, 88 C^c^F_4_S_6_ inbred lines, 13 C^c^F_4_S_7_ inbred lines, seven C^c^F_4_‐DH lines, seven A^r^F_3_S_5_ inbred lines, four A^r^C^c^S_5_ inbred lines and one A^r^C^c^F_2_‐DH line (Table S2). As the development and selection of the C^c^ population started earlier than that of the A^r^ population and A^r^C^c^ genepool, more inbred lines from the C^c^ population were available for genotyping in this study. These lines were developed, selected in the field at Huazhong Agricultural University, Wuhan (semi‐winter growth environment), China, with the same instruments and evaluation methods as described in Xiao *et al*. (2010). To evaluate their phenotype, these lines were planted in Wuhan, China in 2013–2014. Plots for each line were planted in one row with three replicates, with a distance of 30 cm between rows and 15 cm between individuals. Seed quality traits, thousand seed weight and flowering time (the time that 50% of the individuals in the plot were flowering) were tested. Oil content, erucic acid content and glucosinolate content were tested by Foss NIR Systems 5000 using the near‐infrared spectroscopy (NIR) method (Gan *et al*., 2003), and linolenic acid content, linoleic acid content and oleic acid content were tested by NIRS and gas chromatography.

### Representative lines of the parental species of the new‐type *B. napus* used as controls in this study

Fifteen *B. rapa* accessions (coded Br‐1 to Br‐15, 11 of which were used for the synthesis of new‐type *B. napus*) and 14 *B. carinata* accessions (coded Bc‐1 to Bc‐14, 10 of which were used for the synthesis of new‐type *B. napus*) were genotyped as controls using the Illumina Infinium 60K *Brassica* SNP array (Table S2). Most were parents of the new‐type *B. napus* lines, including the *B. napus* cultivar HS3 (Qian *et al*., 2014) as the original *B. napus* parent, and were therefore included as controls for comparison in this study.

The same number (130 accessions) of traditional *B. napus* (Table S2) were collected from different regions of China, France, Germany, Poland, Denmark, Canada, Korea, Japan and Australia, representing different genetic branches of *B. napus* (Xu *et al*., 2016). They were also used as controls in this study for comparison with the new‐type *B. napus* and were coded BnT‐1 to BnT‐130 (Table S2).

### DNA extraction, SNP genotyping, physical positioning of the SNP markers and marker filtering

Genomic DNA from the 130 inbred lines of new‐type *B. napus*, three lines of traditional *B. napus* (BnT128, BnT129 and BnT130) not used in Xu *et al*. (2016) and the accessions of *B. rapa* and *B. carinata* were extracted from bulked young leaf tissue using a DNA extraction Kit (NuClean Plant Genomic DNA Kit, ComWin, CW0531) (Table S2). These lines were genotyped at Huazhong Agricultural University using the *Brassica* 60K Illumina Infinium SNP arrays according to the manufacturer's protocol (Illumina Inc., San Diego, CA, http://www.illumina.com/) followed by clustering, quality control and data calling using the GenomeStudio software (Illumina Inc.), as described in previous studies (Liu *et al*., 2016; Xu *et al*., 2016). The genotypes of 127 traditional *B. napus* were obtained from Xu *et al*. (2016).

The physical position of each SNP marker was assigned according to the top BLAST hits for the probe sequences of each SNP in the array against the reference genome of *B. napus* ‘Darmor‐*bzh*’(version 4.1) (Chalhoub *et al*., 2014) by BlastN using an *e*‐value threshold of *e* ^−10^ (Altschul *et al*., 1990). BLAST matches to multiple loci with the same top e‐value were considered to have multiple positions and classed as unassigned markers without unique positions on the reference genome (Liu *et al*., 2016).

In our study, we used the *Brassica* 60K Illumina Infinium SNP array, which was designed for genotyping cultivars with A/C genomes. However, *B. rapa* and *B. carinata* have only an A genome and only a C genome, respectively, not both, and variation between the *B. rapa* and *B. carinata* A and C genomes and the *B. napus* genome for which the array was designed means that not all markers on the array were successfully amplified in *B. rapa* and *B. carinata*. Consequently, a high rate of missing values for the accessions of *B. rapa* (average 42.8%) and *B. carinata* (average 31.6%) was generated. Therefore, we ignored the percentage of missing values in these species for calculation. All of the other investigated samples should have less than 15% missing values in general across all SNPs (the whole 52 157 markers). Considering the potential for actual missing chromosome segments and heterozygosity in new‐type *B. napus*, we first filtered those markers with ≥20% missing values in the subpopulations of new‐type *B. napus* and traditional *B. napus*. Subsequently, we calculated the gene diversity, genetic distance, genetic clustering and *F*_ST_ within and between the subpopulations of new‐type *B. napus* (BnN), traditional *B. napus* (BnT), *B. rapa* (Br), *B. carinata* (Bc) and the parental *B. napus* cultivar of new‐type *B. napus*, HS3. For the LD and haplotype block analysis, we further filtered out those makers with minor allele frequency (MAF) ≤5%, more than 20% heterozygosity, and only used markers with unique positions on the A and C genomes of *B. napus* as determined by BLAST analysis following the methods of Liu *et al*. (2016) and Qian *et al*. (2014). This approach allowed us to compare the LD and haplotype results from new‐type *B. napus* with that of traditional *B. napus* by establishing similar filtering parameters.

### Genetic diversity, genetic distance, *F*_ST_ and population structure analysis

The polymorphism information content (PIC) and genetic diversity of the SNP markers, Nei's genetic distance (Nei and Takezaki, 1983) and *F*_ST_ within and among the populations were estimated using the software PowerMarker Version 3.25 (Liu and Muse, 2005) and Arlequin ver. 3.5.1.2 (Excoffier *et al*., 2007). The cluster dendrogram of the relationships between investigated lines/accessions was constructed using *MEGA* version 6 (Tamura *et al*., 2013) with the genetic distance matrix. Principal component analysis (PCA) was performed with Tassel version 5 (Bradbury *et al*., 2007).

### Linkage disequilibrium and haplotype block analysis

The pattern of linkage disequilibrium (LD) on each chromosome and across the A and C subgenome was estimated based on the parameter *r* ^2^ of all pairwise‐filtered SNPs with unique physical positions and were calculated using the software TASSEL version 5 (Bradbury *et al*., 2007). Heterozygous SNPs were set as missing. LD decay of the new‐type *B. napus* population was estimated based on the corresponding physical distance of pairwise SNPs with the value of *r* ^2^ decreased to half of the maximum, 0.2 and 0.1, and compared with the results of traditional *B. napus* (Liu *et al*., 2016). The same marker data set for LD analysis was used for analysing the haplotype block structure across the population by Haploview v4.2 (Barrett *et al*., 2005). Haplotype blocks were defined according to ‘strong LD’ with an upper 95% confidence bound of D’ above 0.70–0.98 (Gabriel *et al*., 2002; Qian *et al*., 2014; Sun *et al*., 2016). Haplotype blocks of the new‐type *B. napus* population were defined based on the same methods and compared with those of traditional *B. napus* reported in Sun *et al*. (2016).

### Detection of identity by descent and introgressions originating from the original parents of the new‐type *B. napus* lines

Considering the specific genetic diversity of *B. rapa* and *B. carinata*, we used the whole marker data set filtered for genetic diversity analysis to estimate exotic introgressions in the new‐type *B. napus* lines. Exotic introgressions in each line of new‐type *B. napus* were estimated according to the average possibility that the alleles originated from the parents across the genome (Zou *et al*., 2010). Using markers with unique physical positions on the AC genome from BLAST analysis, we analysed the identity by descent (IBD) of each chromosome inherited from the original *B. napus* parent HS3, parental *B. rapa* and *B. carinata* accessions in the genome of new‐type *B. napus*, using the software fastIBD (Beagle version 4.0) with a *P* value (*E*) < 10^−9^ for linked markers (Browning and Browning, 2011).

### Detection of deletions and duplications in the new‐type *B. napus* genomes

According to the normalized ratio of the fluorescence intensity of the hybridization signals for each of the markers on the SNP array chip, the regions of putative deletion and duplication were determined according to the method described in Grandke *et al*. (2016) using the R package GSRC 1.04 (Genome Structure Rearrangement Calling in Genomes with High Synteny) and the markers with unique positions on the A and C genomes of *B. napus*. We also analysed putative deletion/duplication events by identifying regions with contiguous missing markers (with at least five markers used as the cut‐off to identify a deletion/duplication).

### Selective sweep analysis

The nucleotide diversity of the markers in the new type *B. napus* lines and traditional *B. napus* lines were calculated using TASSEL version 5 (Bradbury *et al*., 2007). Estimation of the SNP and population‐specific *F*_ST_ was based on the pure drift model (Nicholson *et al*., 2002), following the procedure described by Porto‐Neto *et al*. (2013). Selection statistics (Tajima's *D*, a measure of selection in the genome) was calculated using the software TASSEL version 5 (Bradbury *et al*., 2007). A sliding‐window approach (100‐kb windows sliding in 10‐kb steps) was applied to quantify the polymorphism levels (θ_π_, pairwise nucleotide variation as a measure of variability), genetic differentiation (*F*_ST_) and selection statistics (Tajima's *D*) of the new‐type *B. napus* and traditional *B. napus*. The mean was set as zero when the 100‐kb windows contained <3 SNP markers. Finally, to detect regions with significant signatures of selective sweeps in the new‐type *B. napus*, the distribution of the θ_π_ ratios (θ_π,__BnT_/θ_π,__BnN_) and *F*_ST_ values between the two populations (the new‐type *B. napus* and traditional *B. napus*), as well as Tajima's *D* values in the new‐type *B. napus* lines, was considered simultaneously. The regions with significantly high θ_π_ ratios (the 5% right tail) and significantly high *F*_ST_ values (the 5% right tail) of the empirical distribution were treated as regions with strong selective sweeps in the new‐type *B. napus* (Li *et al*., 2013). The regions with significantly high θ_π_ ratios (the 5% right tails) of the empirical distribution and negative Tajima's *D* values were treated as putatively selected regions in the new‐type *B. napus*.

## Author contributions

JZ and DDH performed the research, analysed the data and wrote the manuscript; ASM, XQS, XHW, NW, FG, RJS and JLM contributed to methods and tools. MW and SHC contributed to plant growth and phenotyping. JLM contributed to plant materials. JZ designed the research. All authors revised, read and approved the final manuscript.

## Competing interests

The authors declare that they have no competing interests.

## Supporting information


**Figure S1** Linkage disequilibrium and haplotype blocks of the new‐type *Brassica napus* population across the whole genome, and its comparison with traditional *Brassica napus*.

**Figure S2** Distribution of the haplotype blocks in the new‐type *Brassica napus* population.

**Figure S3** Distribution of identity‐by‐descent blocks (IBD) originating from the parents of the new‐type *Brassica napus* population.

**Table S1** Name and geographical origin of parental lines involved in the development of the new‐type *B. napus*.

**Table S2** New‐type *Brassica napus* lines and accessions of the parental species investigated in this study and their characteristics.

**Table S3** Polymorphism within and among the population of new‐type *Brassica napus* and its parental species as evaluated by the Illumina Infinium *Brassica* 60K SNP array.

**Table S4** Haplotype blocks >50 kb identified in the new‐type *Brassica napus* and traditional *Brassica napus* populations.

**Table S5** Identity‐by‐descent (IBD) segments originating from *Brassica napus ‘*HS3’ within the new‐type *Brassica napus* population.

**Table S6** Deletion and duplication regions >50 kb in size in the new‐type *Brassica napus* population.

**Table S7** Selected regions identified in the population of new‐type *Brassica napus* and traditional *Brassica napus*.
