# Supplementary material for: Genetic changes in a novel breeding population of Brassica napus synthesized from hundreds of crosses between B. rapa and B. carinata
Source: Plant Biotechnol J. 2017 Aug 16;16(2):507–19. doi: 10.1111/pbi.12791 (PMC5811809; doi:10.1111/pbi.12791)
Supplement: Supplementary file 1 — Figure S1 Linkage disequilibrium and haplotype blocks of the new‐type Brassica napus population across the whole genome, and its comparison with traditional Brassica napus. [file PBI-16-507-s005.pdf]

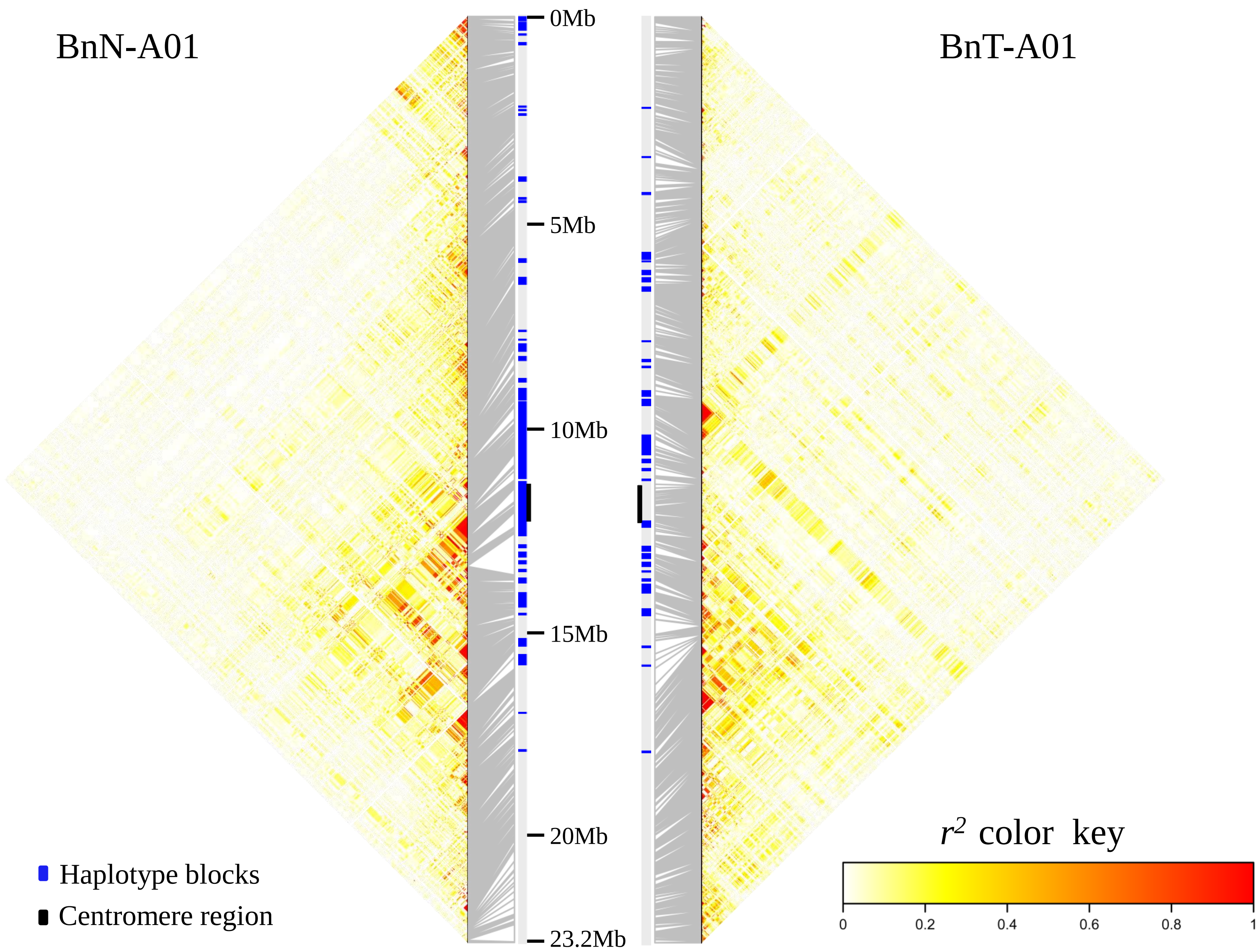
BnN-A01
BnT-A01
0Mb
5Mb
10Mb
15Mb
20Mb
23.2Mb
Haplotype blocks
Centromere region
r2 color key
0
0.2
0.4
0.6
0.8
1

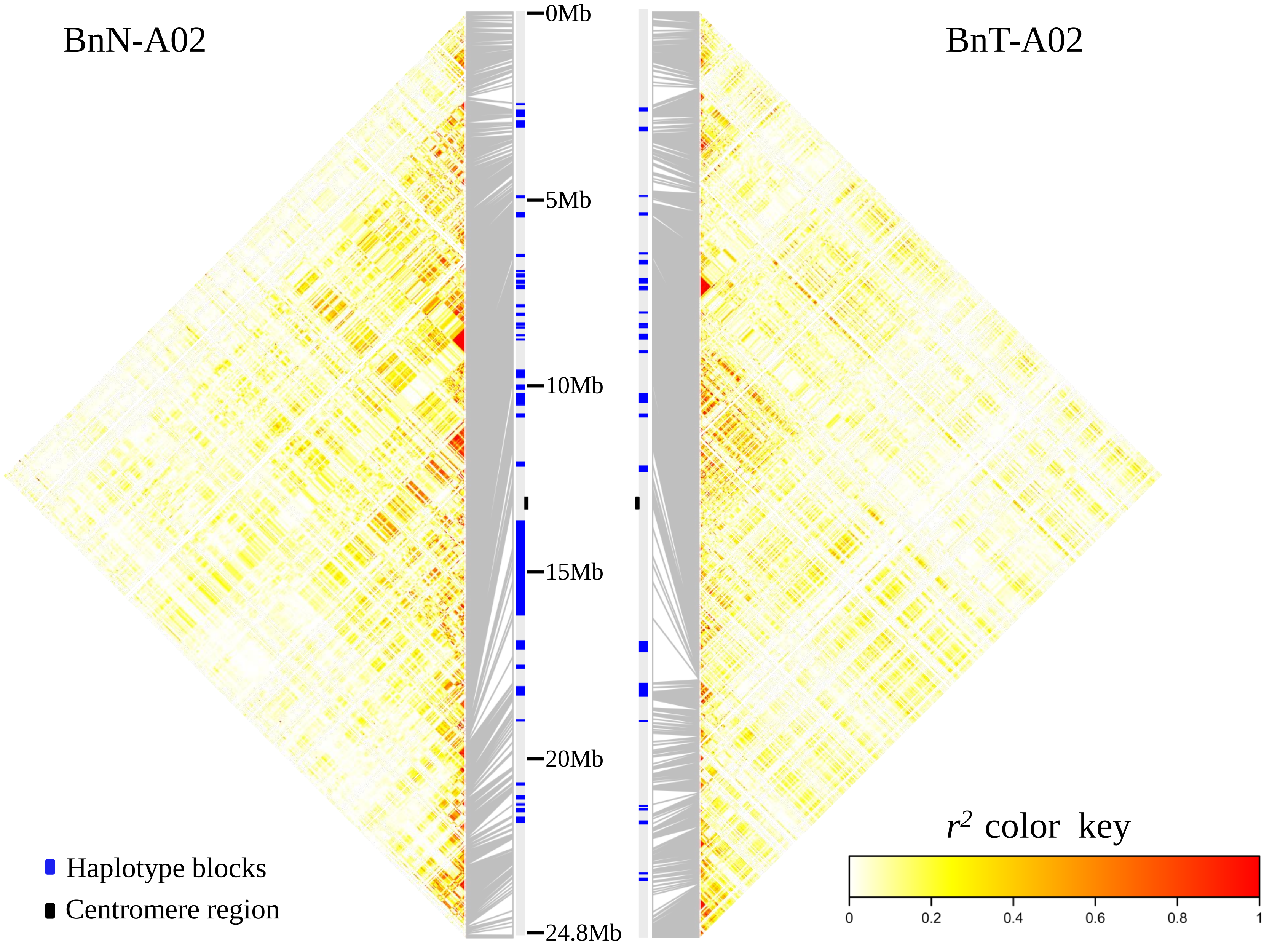

BnN-A02
BnT-A02
0Mb
5Mb
10Mb
15Mb
20Mb
24.8Mb
Haplotype blocks
Centromere region
$r^2$ color key
0
0.2
0.4
0.6
0.8
1

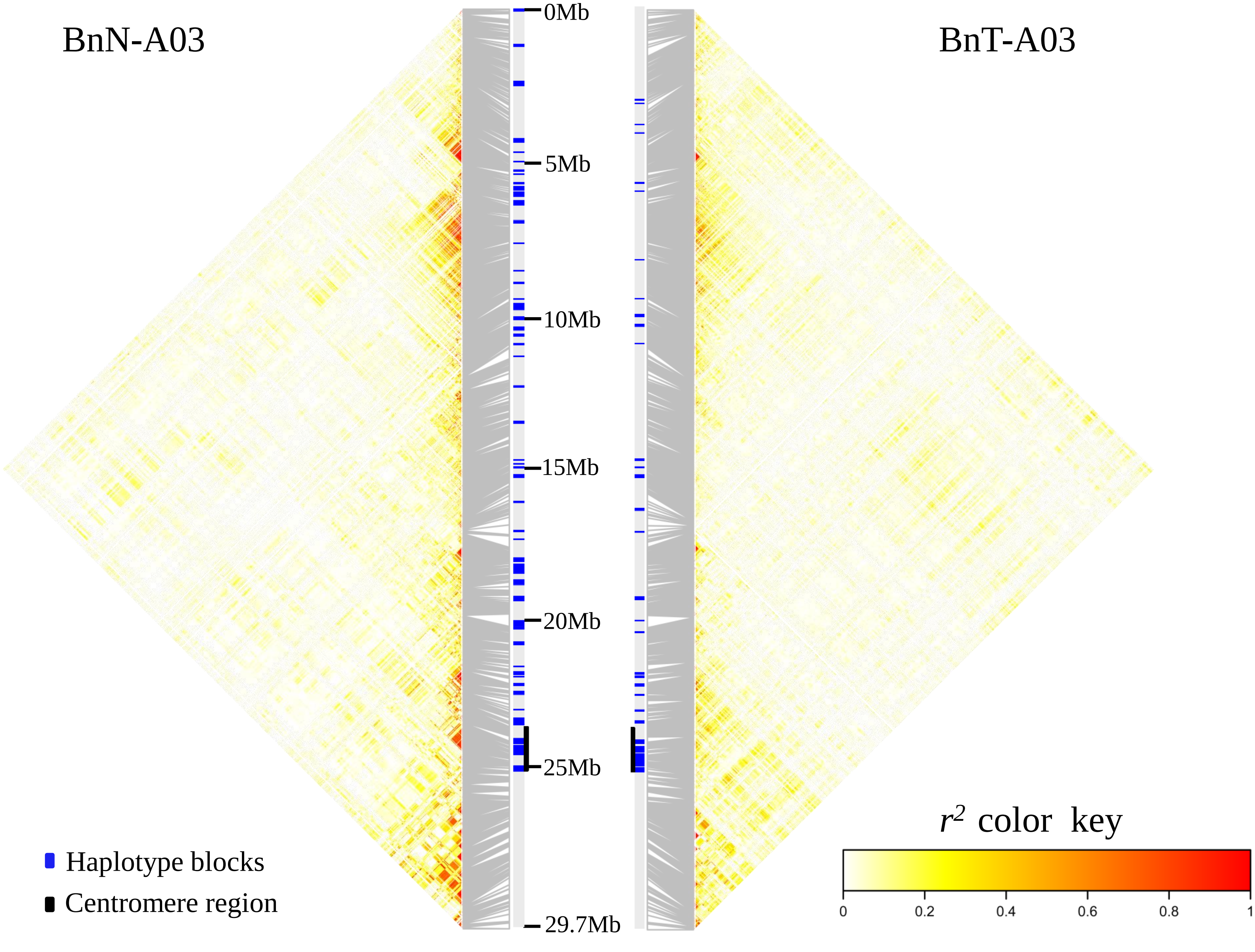
BnN-A03
BnT-A03
0Mb
5Mb
10Mb
15Mb
20Mb
25Mb
29.7Mb
Haplotype blocks
Centromere region
r2 color key
0
0.2
0.4
0.6
0.8
1

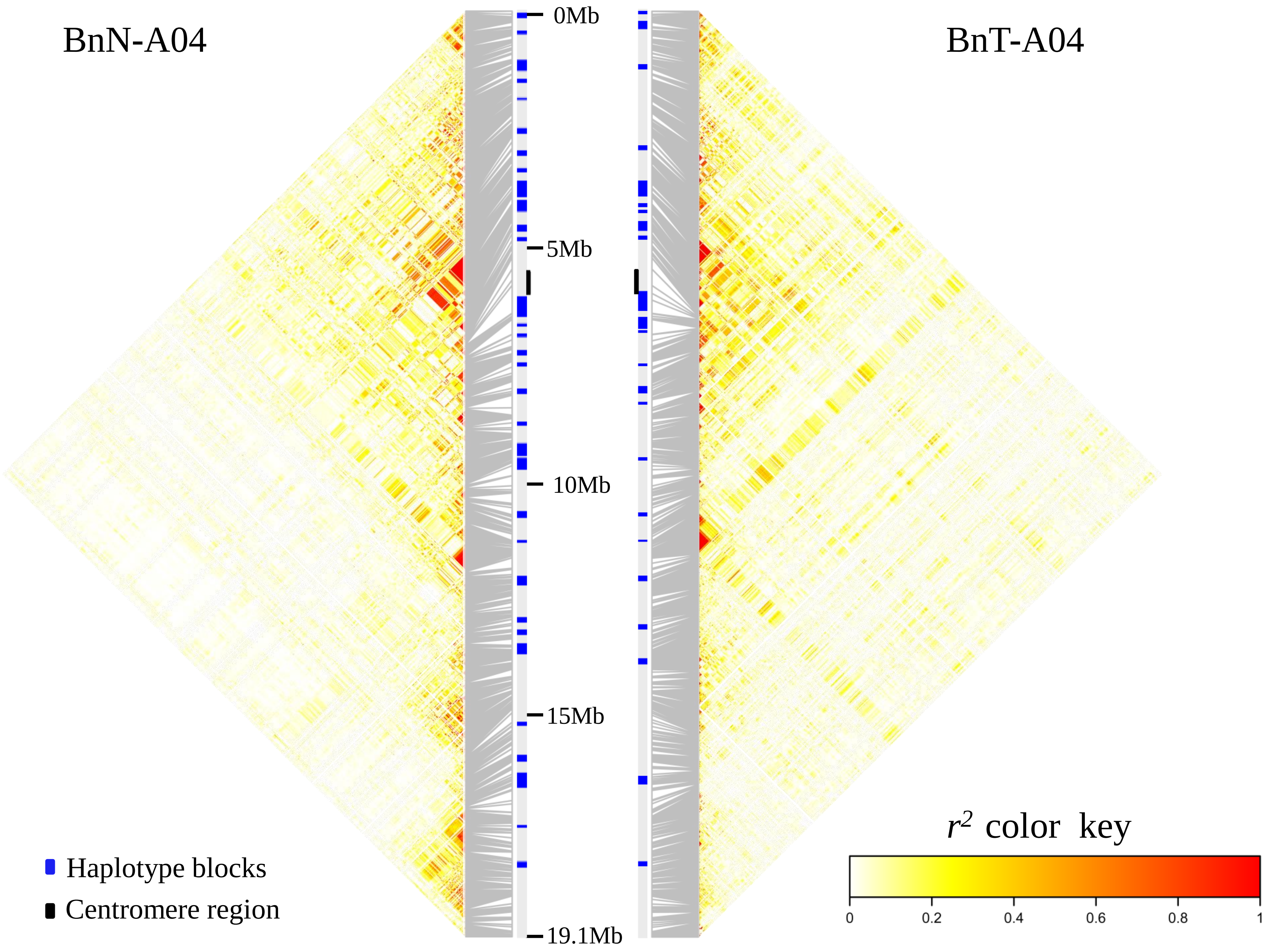

BnN-A04
BnT-A04
0Mb
5Mb
10Mb
15Mb
19.1Mb
Haplotype blocks
Centromere region
$r^2$ color key
0
0.2
0.4
0.6
0.8
1

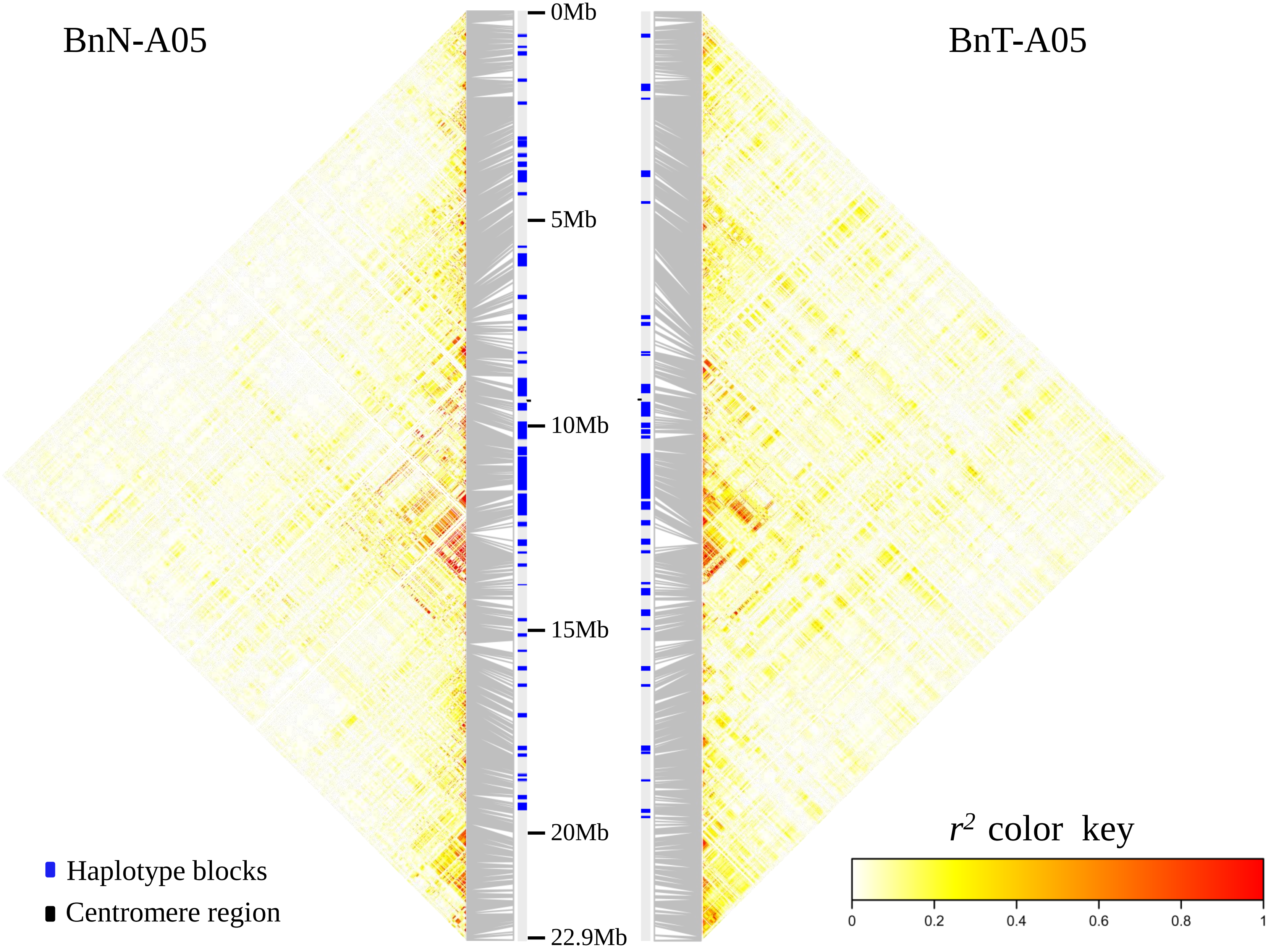
BnN-A05
BnT-A05
0Mb
5Mb
10Mb
15Mb
20Mb
22.9Mb
Haplotype blocks
Centromere region
$r^2$ color key
0
0.2
0.4
0.6
0.8
1

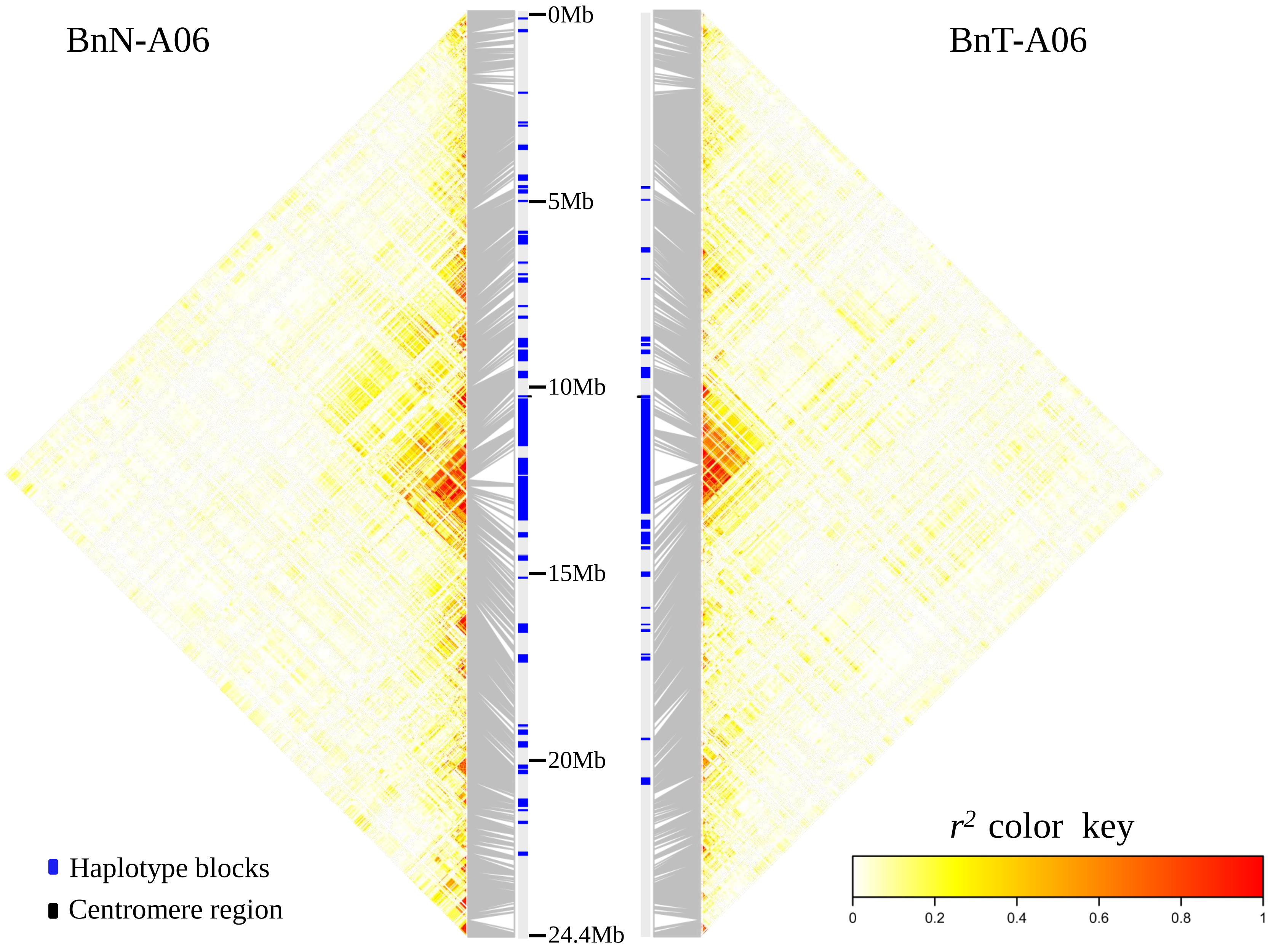
BnN-A06
BnT-A06
0Mb
5Mb
10Mb
15Mb
20Mb
24.4Mb
Haplotype blocks
Centromere region
r2 color key
0
0.2
0.4
0.6
0.8
1

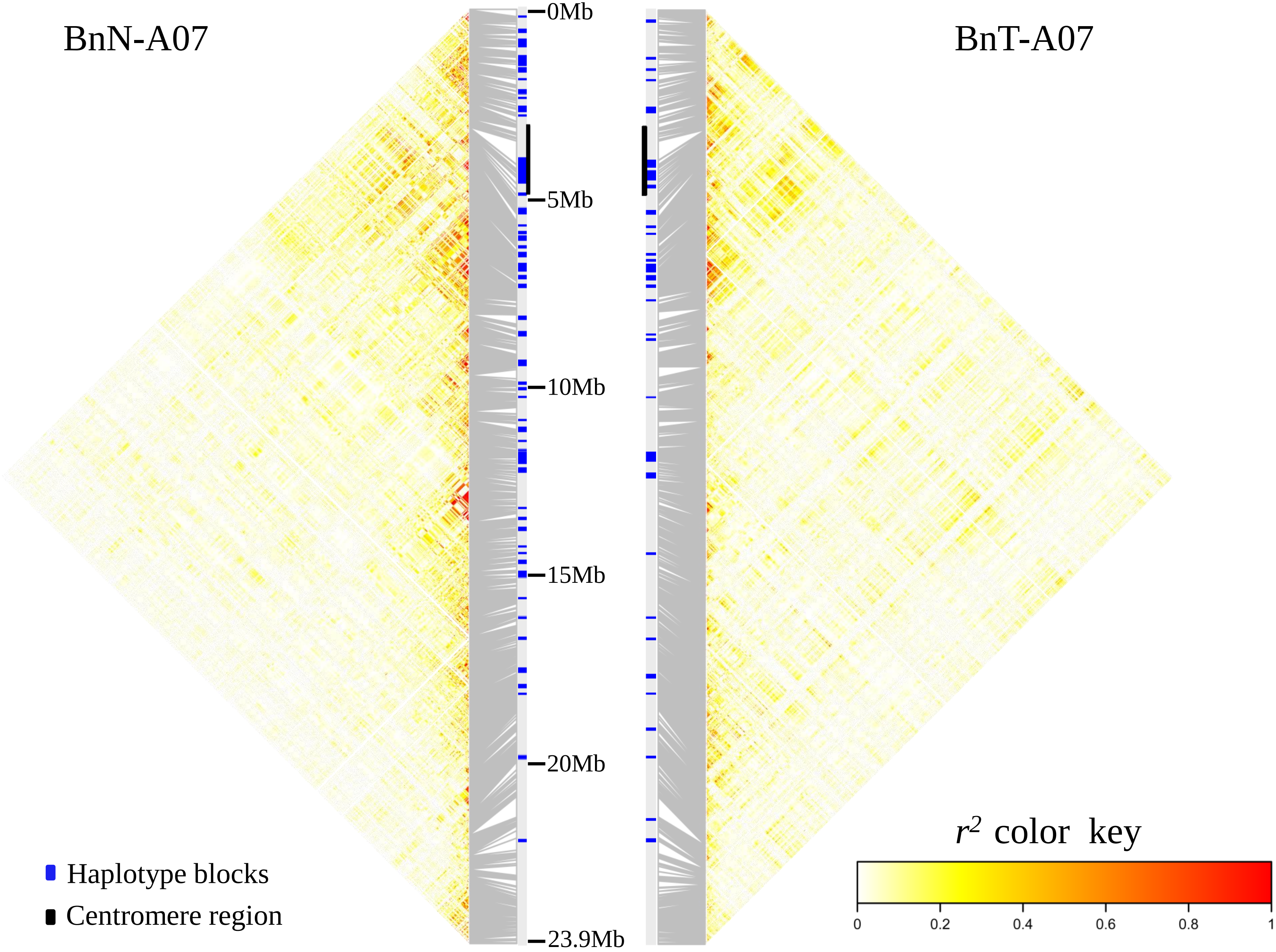
BnN-A07
BnT-A07
0Mb
5Mb
10Mb
15Mb
20Mb
23.9Mb
Haplotype blocks
Centromere region
$r^2$ color key
0
0.2
0.4
0.6
0.8
1

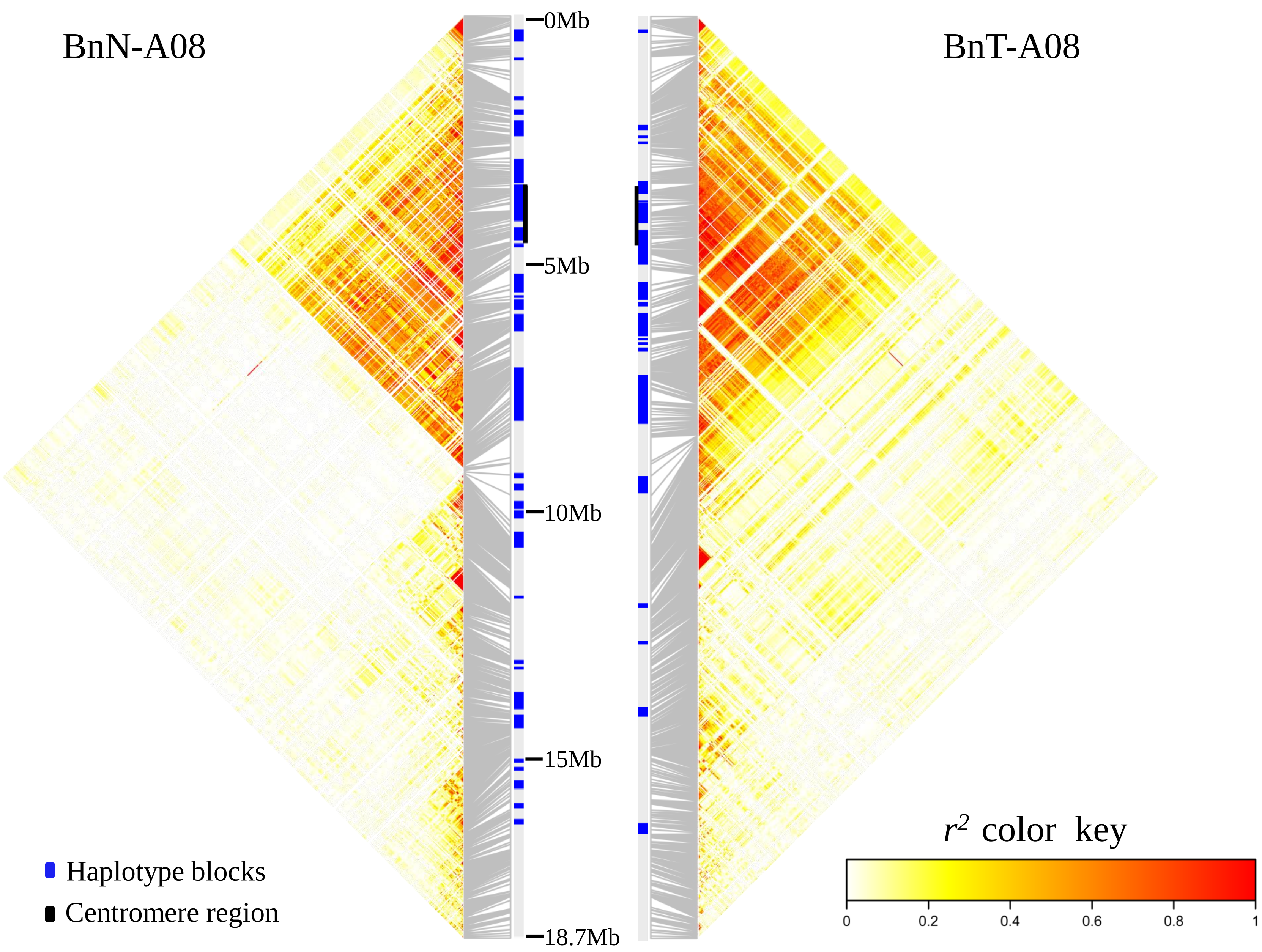

BnN-A08
BnT-A08
0Mb
5Mb
10Mb
15Mb
18.7Mb
Haplotype blocks
Centromere region
$r^2$ color key
0
0.2
0.4
0.6
0.8
1

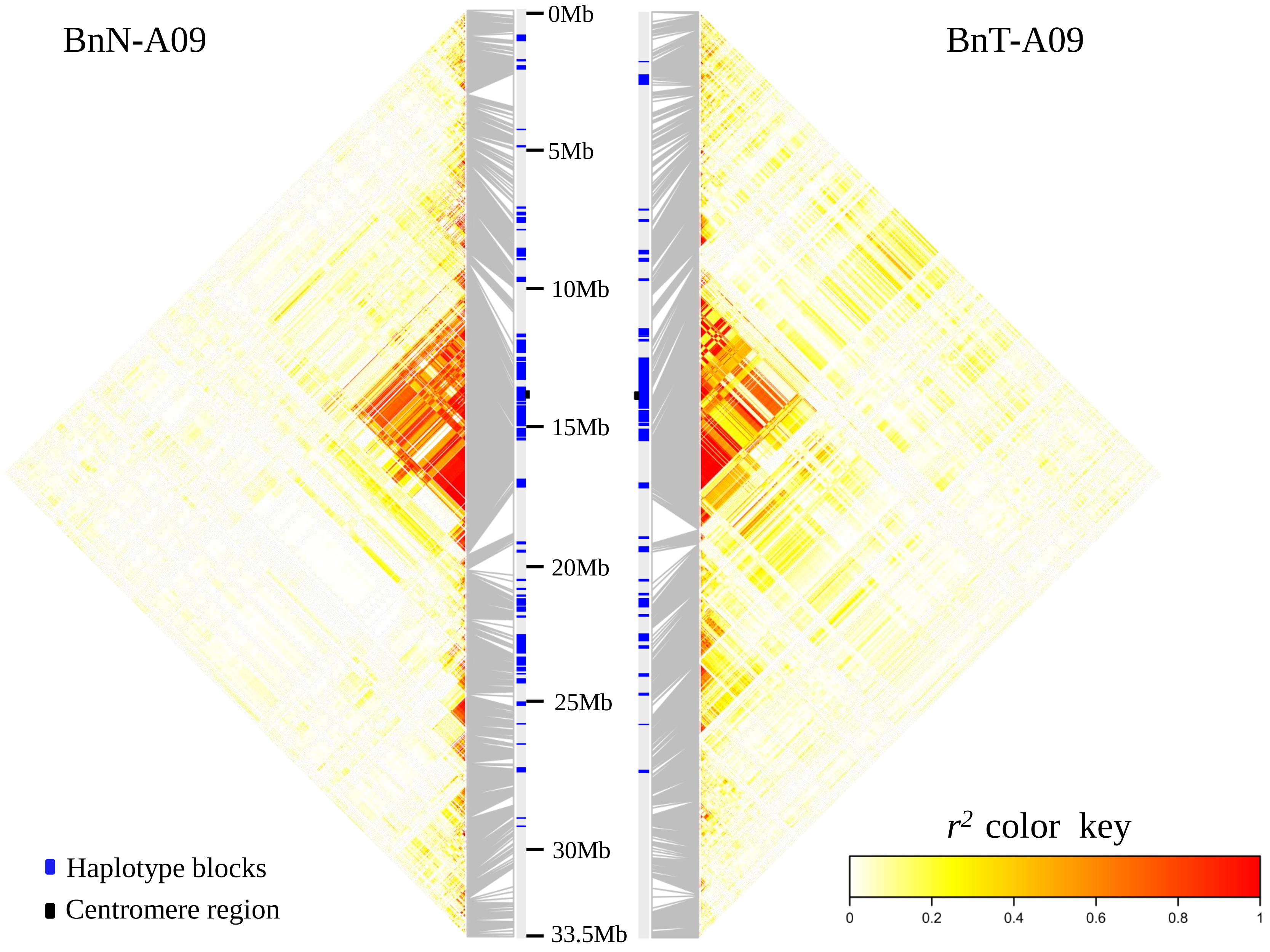
BnN-A09
BnT-A09
0Mb
5Mb
10Mb
15Mb
20Mb
25Mb
30Mb
33.5Mb
Haplotype blocks
Centromere region
r2 color key
0
0.2
0.4
0.6
0.8
1

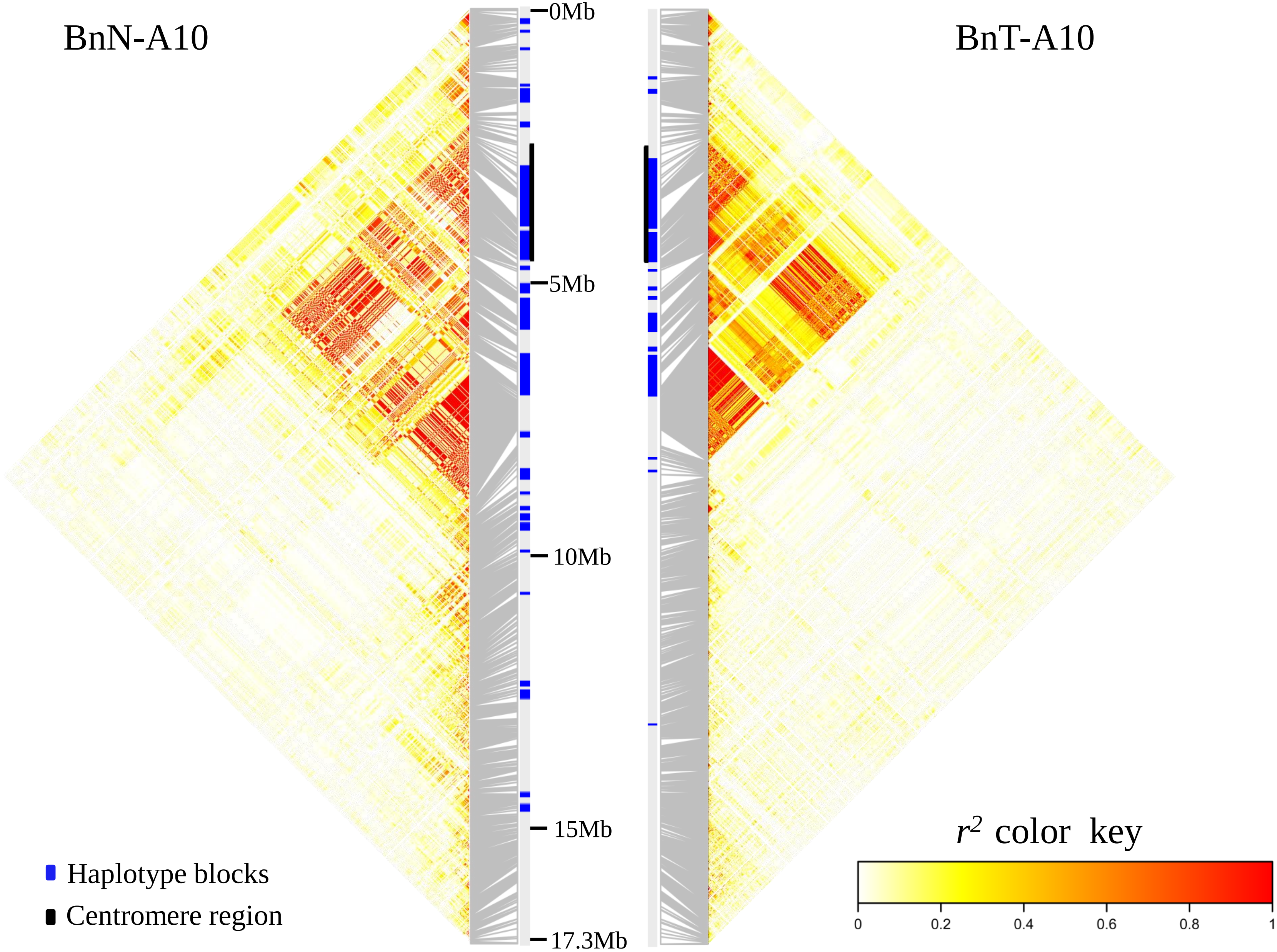
BnN-A10
BnT-A10
0Mb
5Mb
10Mb
15Mb
17.3Mb
Haplotype blocks
Centromere region
$r^2$ color key
0
0.2
0.4
0.6
0.8
1

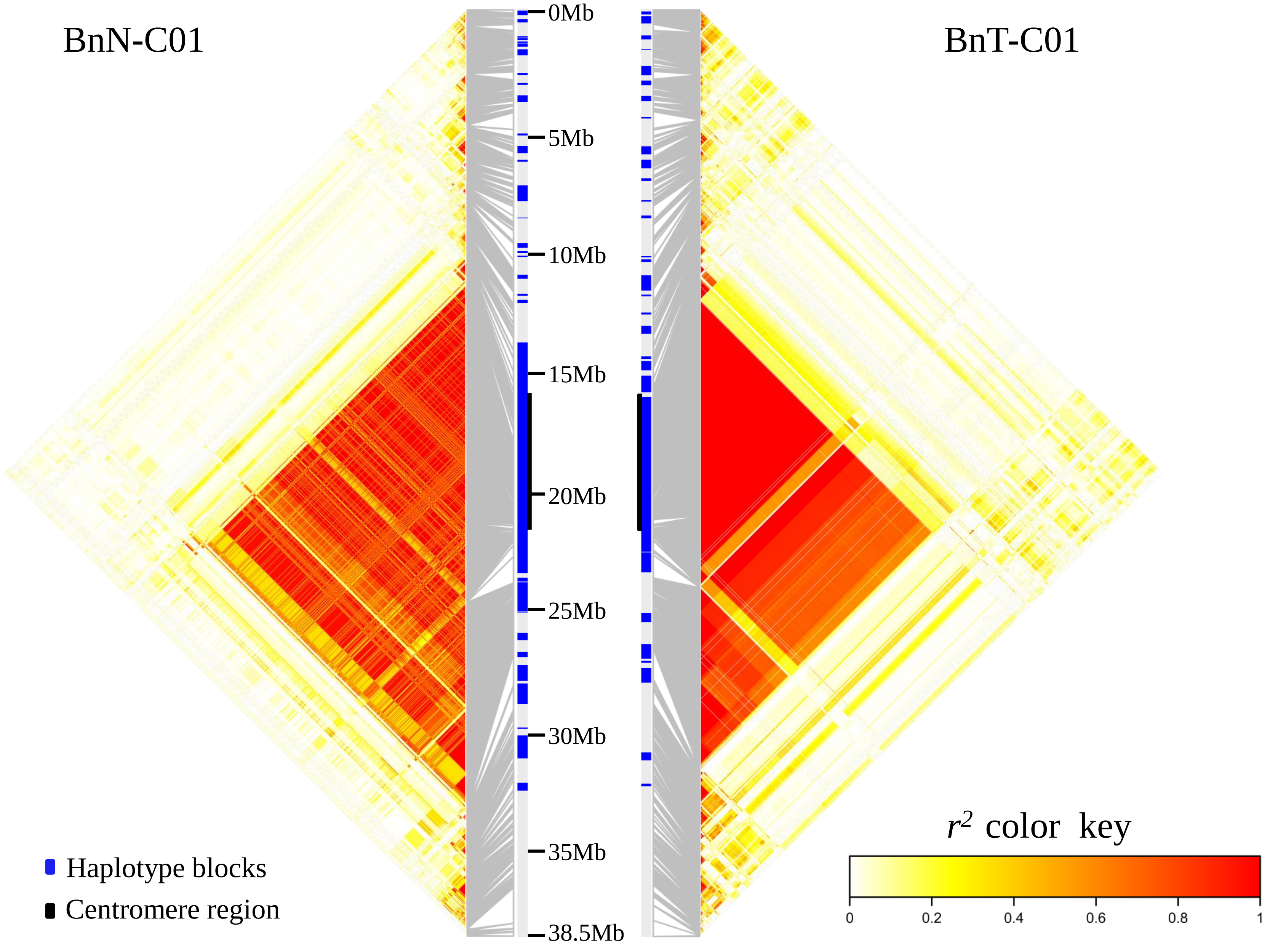
BnN-C01
BnT-C01
0Mb
5Mb
10Mb
15Mb
20Mb
25Mb
30Mb
35Mb
38.5Mb
Haplotype blocks
Centromere region
$r^2$ color key
0
0.2
0.4
0.6
0.8
1

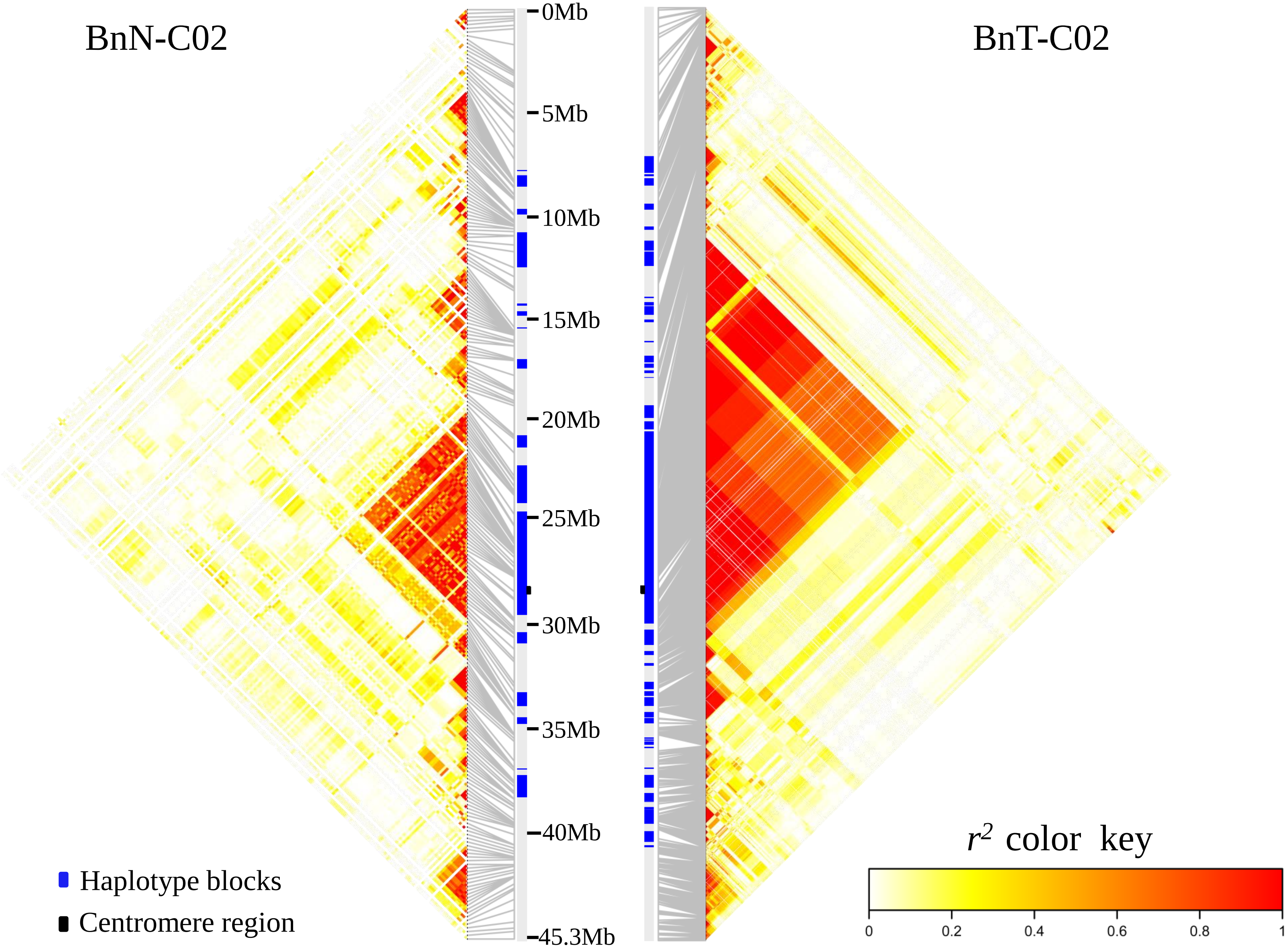
BnN-C02
BnT-C02
0Mb
5Mb
10Mb
15Mb
20Mb
25Mb
30Mb
35Mb
40Mb
45.3Mb
Haplotype blocks
Centromere region
r2 color key
0
0.2
0.4
0.6
0.8
1

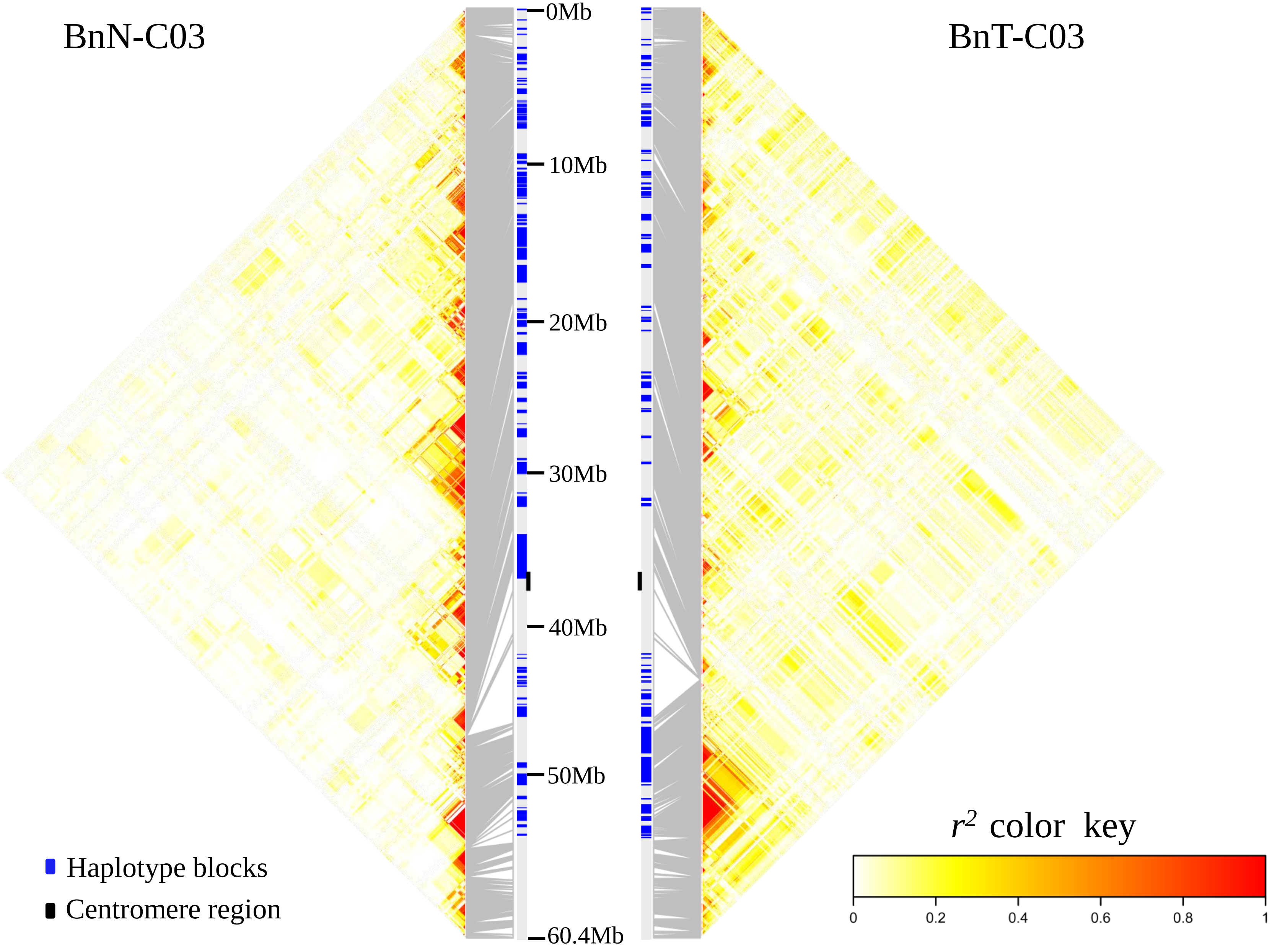
BnN-C03
BnT-C03
0Mb
10Mb
20Mb
30Mb
40Mb
50Mb
60.4Mb
Haplotype blocks
Centromere region
$r^2$ color key
0
0.2
0.4
0.6
0.8
1

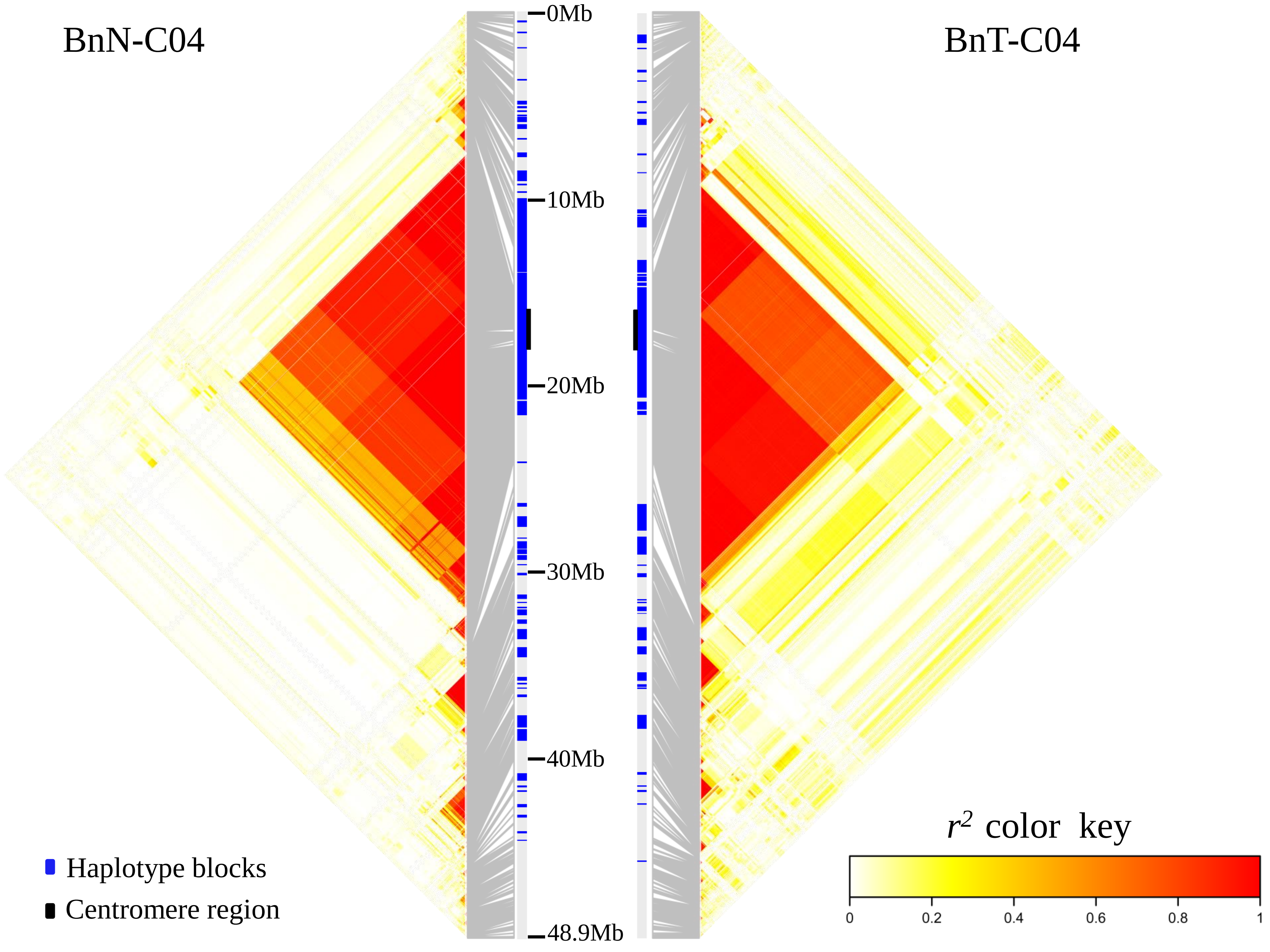
BnN-C04
BnT-C04
0Mb
10Mb
20Mb
30Mb
40Mb
48.9Mb
Haplotype blocks
Centromere region
$r^2$ color key
0
0.2
0.4
0.6
0.8
1

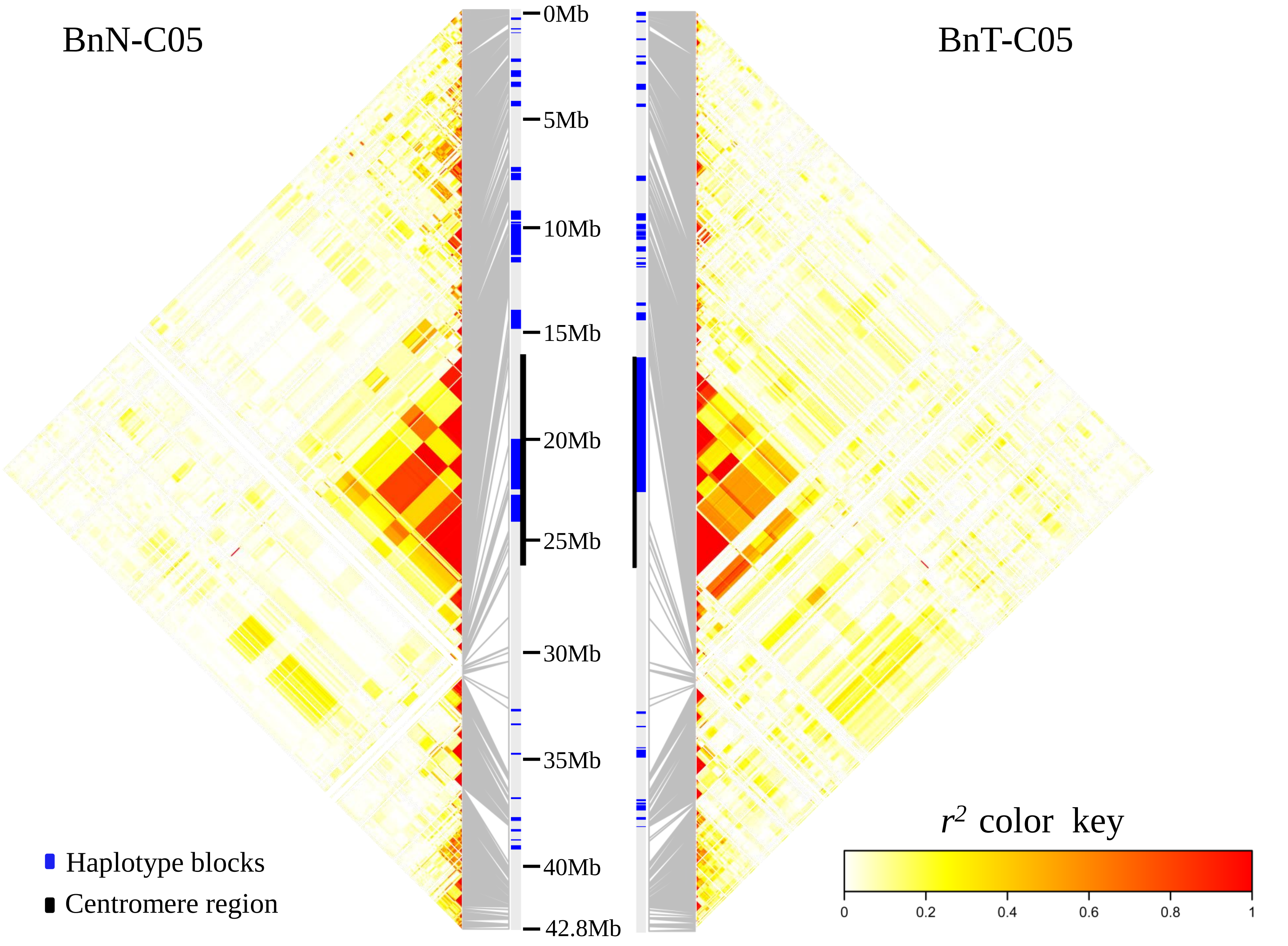
BnN-C05
BnT-C05
0Mb
5Mb
10Mb
15Mb
20Mb
25Mb
30Mb
35Mb
40Mb
42.8Mb
Haplotype blocks
Centromere region
$r^2$ color key
0
0.2
0.4
0.6
0.8
1

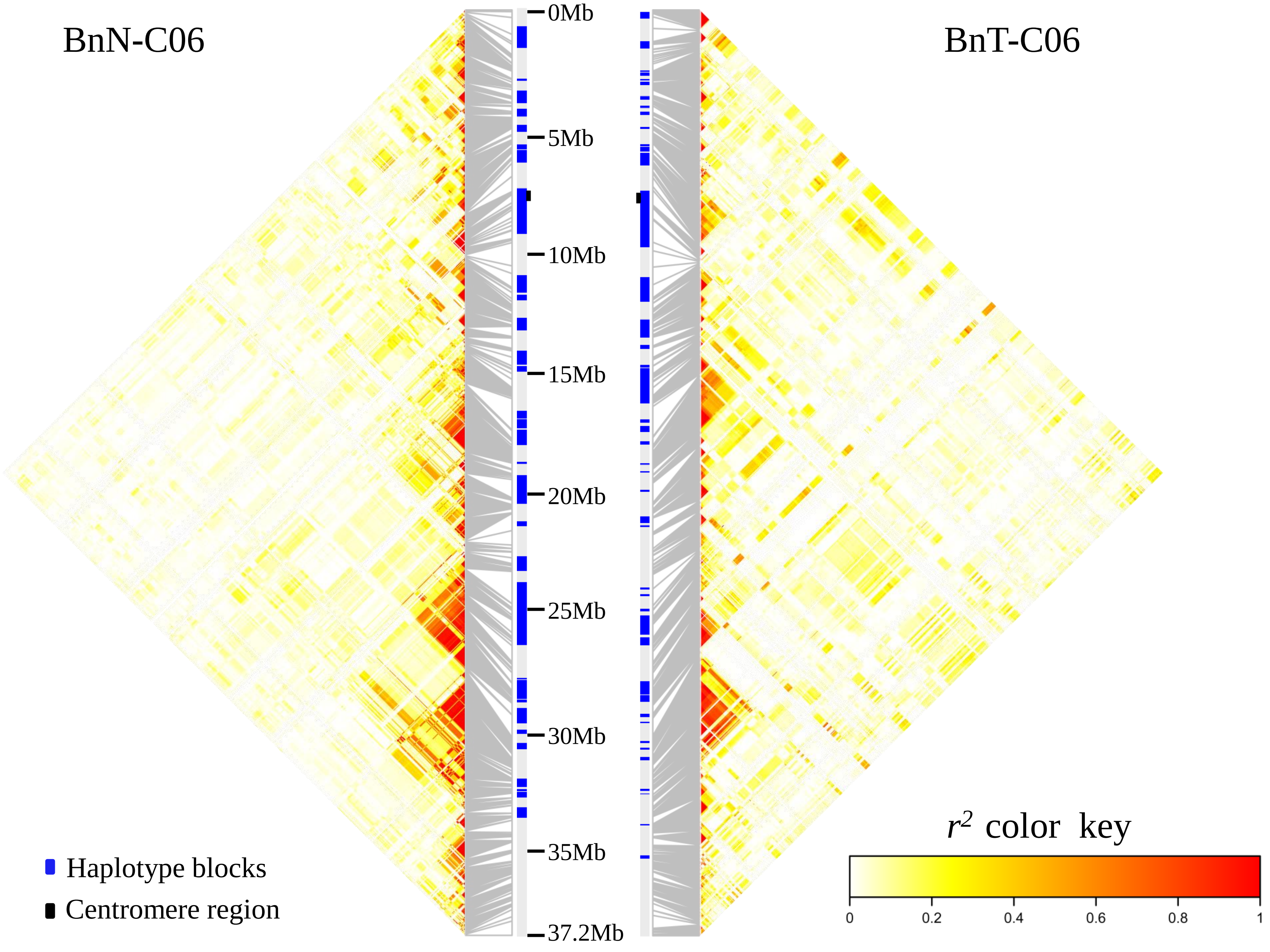

BnN-C06
BnT-C06
0Mb
5Mb
10Mb
15Mb
20Mb
25Mb
30Mb
35Mb
37.2Mb
Haplotype blocks
Centromere region
r2 color key
0
0.2
0.4
0.6
0.8
1

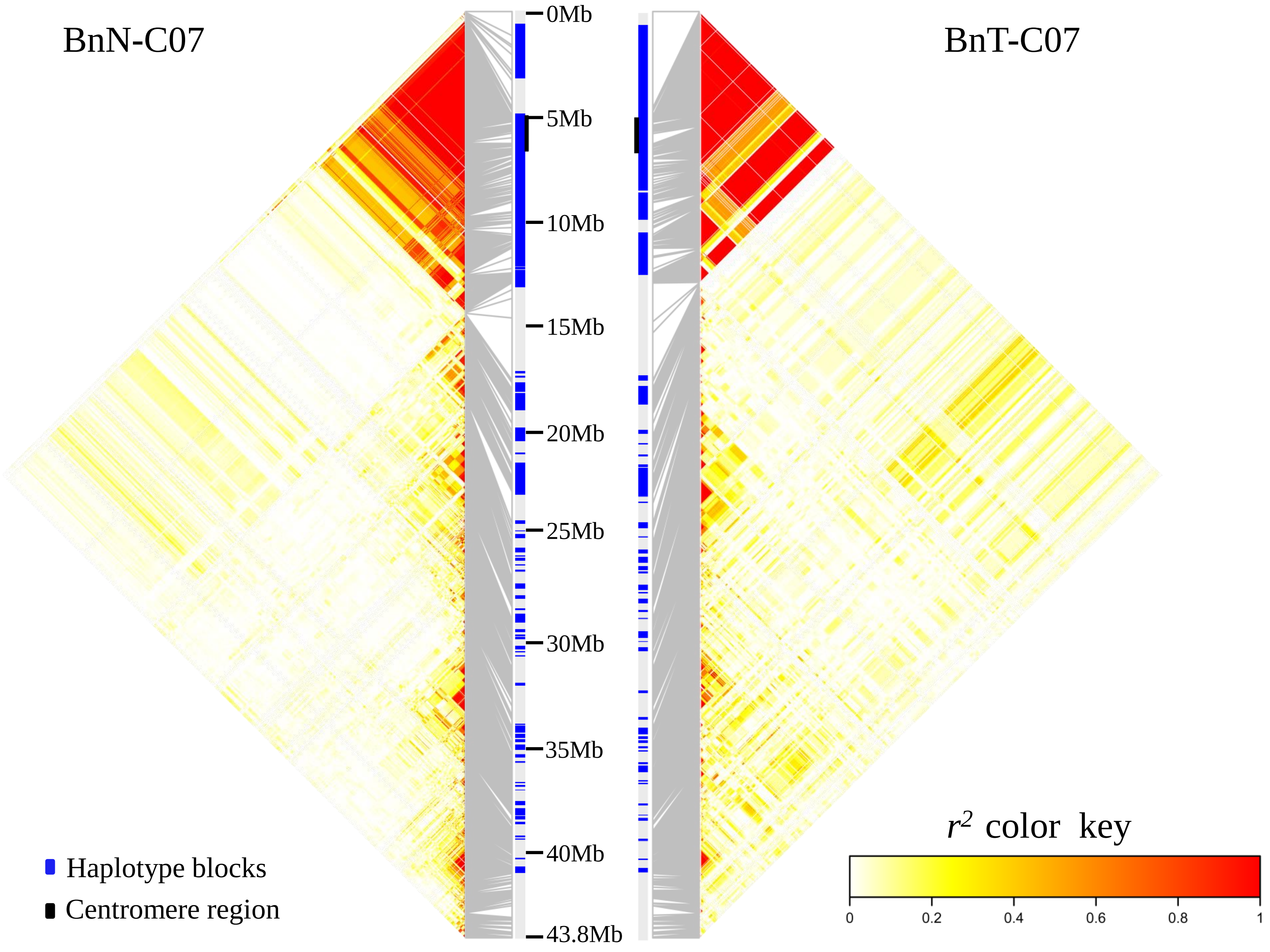

BnN-C07
BnT-C07
0Mb
5Mb
10Mb
15Mb
20Mb
25Mb
30Mb
35Mb
40Mb
43.8Mb
Haplotype blocks
Centromere region
$r^2$ color key
0
0.2
0.4
0.6
0.8
1

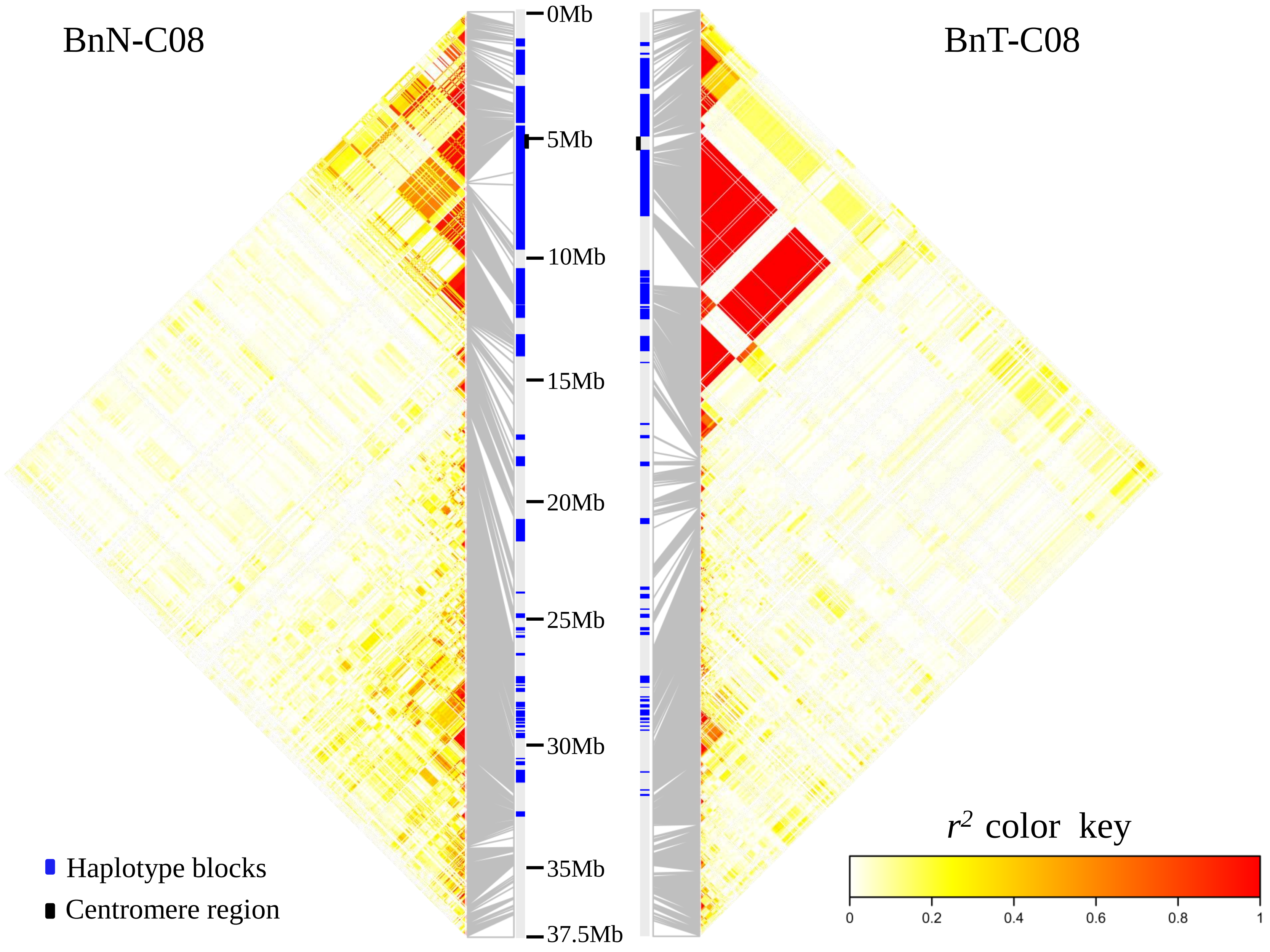
BnN-C08
BnT-C08
0Mb
5Mb
10Mb
15Mb
20Mb
25Mb
30Mb
35Mb
37.5Mb
Haplotype blocks
Centromere region
$r^2$ color key
0
0.2
0.4
0.6
0.8
1

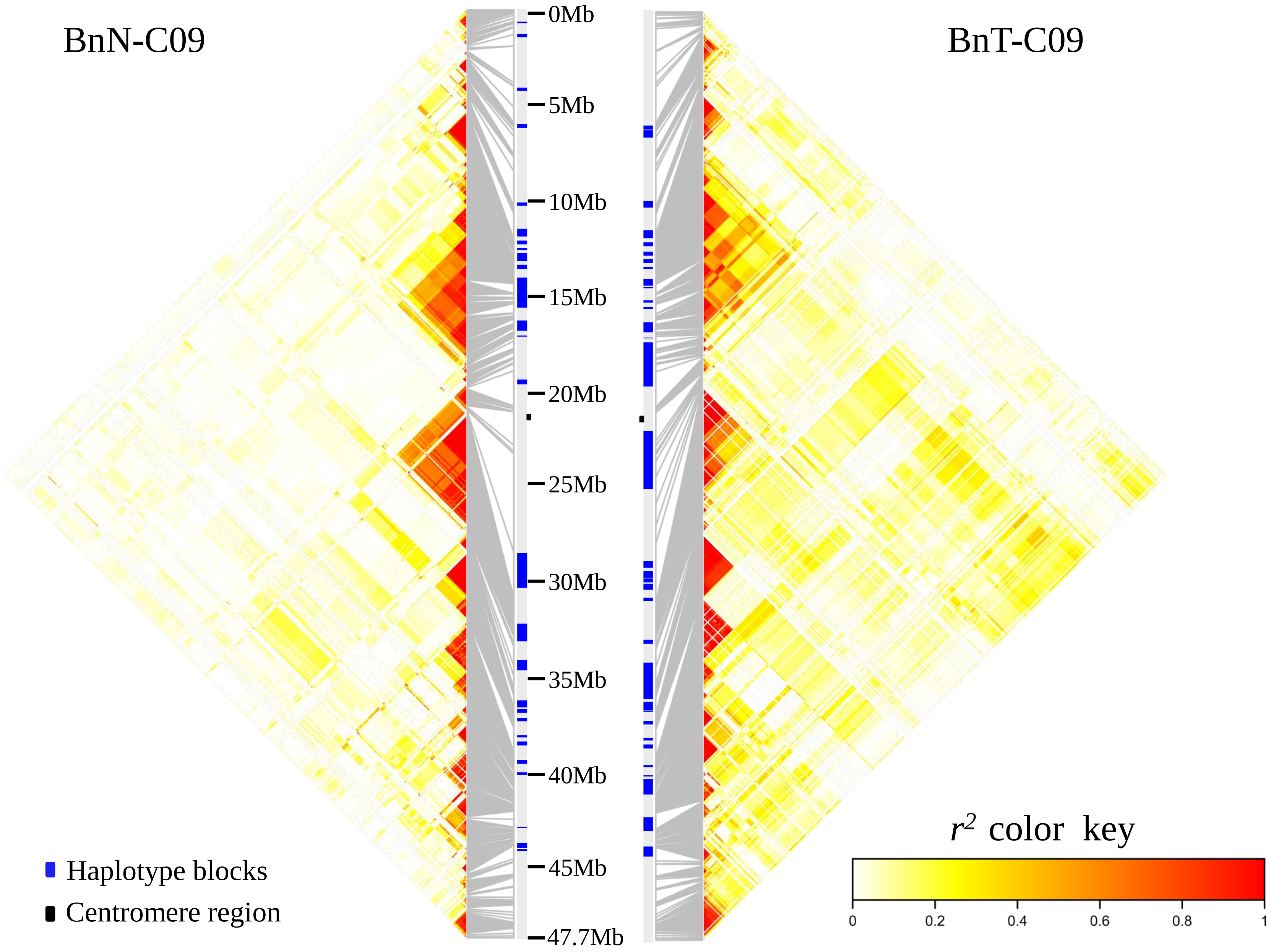

BnN-C09
BnT-C09
0Mb
5Mb
10Mb
15Mb
20Mb
25Mb
30Mb
35Mb
40Mb
45Mb
47.7Mb
Haplotype blocks
Centromere region
r2 color key
0
0.2
0.4
0.6
0.8
1
