# Supplementary figures and images for: Genetic changes in a novel breeding population of Brassica napus synthesized from hundreds of crosses between B. rapa and B. carinata
Source: Plant Biotechnol J. 2017 Aug 16;16(2):507–19. doi: 10.1111/pbi.12791 (PMC5811809; doi:10.1111/pbi.12791)

**A**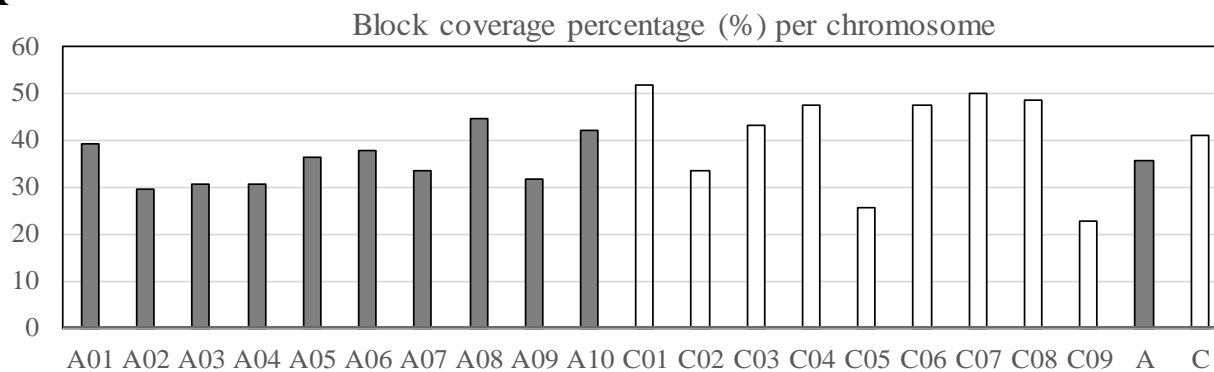**B**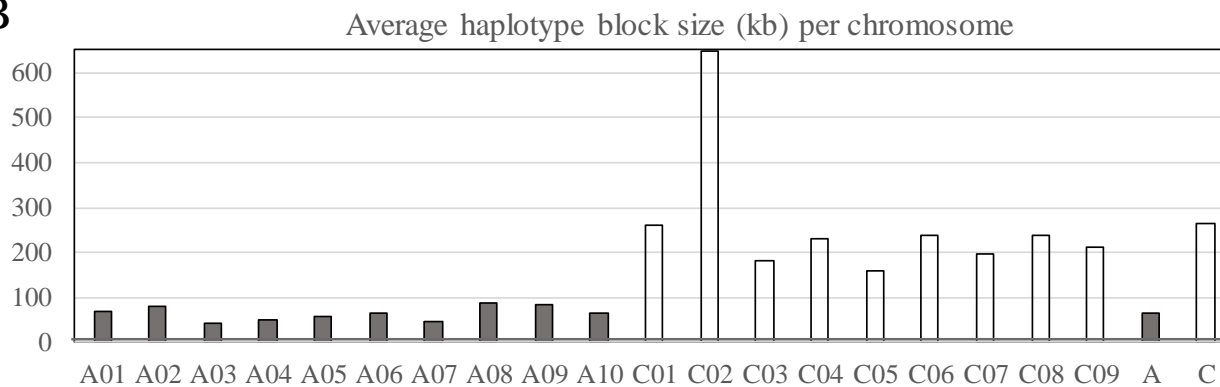**C**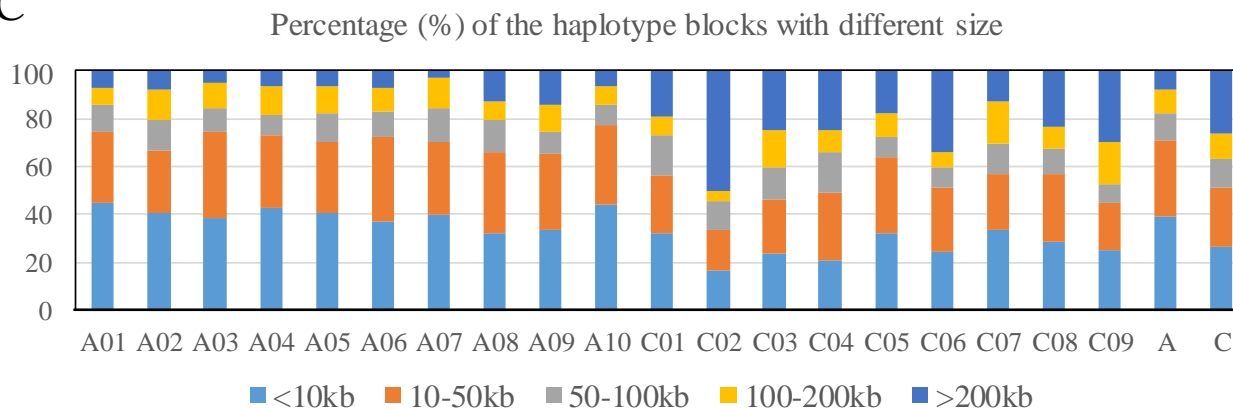

Supplement: Supplementary file 2 — Figure S2 Distribution of the haplotype blocks in the new‐type Brassica napus population. [file PBI-16-507-s008.pdf]
