# Supplementary material for: Genetic changes in a novel breeding population of Brassica napus synthesized from hundreds of crosses between B. rapa and B. carinata
Source: Plant Biotechnol J. 2017 Aug 16;16(2):507–19. doi: 10.1111/pbi.12791 (PMC5811809; doi:10.1111/pbi.12791)
Supplement: Supplementary file 3 — Figure S3 Distribution of identity‐by‐descent blocks (IBD) originating from the parents of the new‐type Brassica napus population. [file PBI-16-507-s001.pdf]

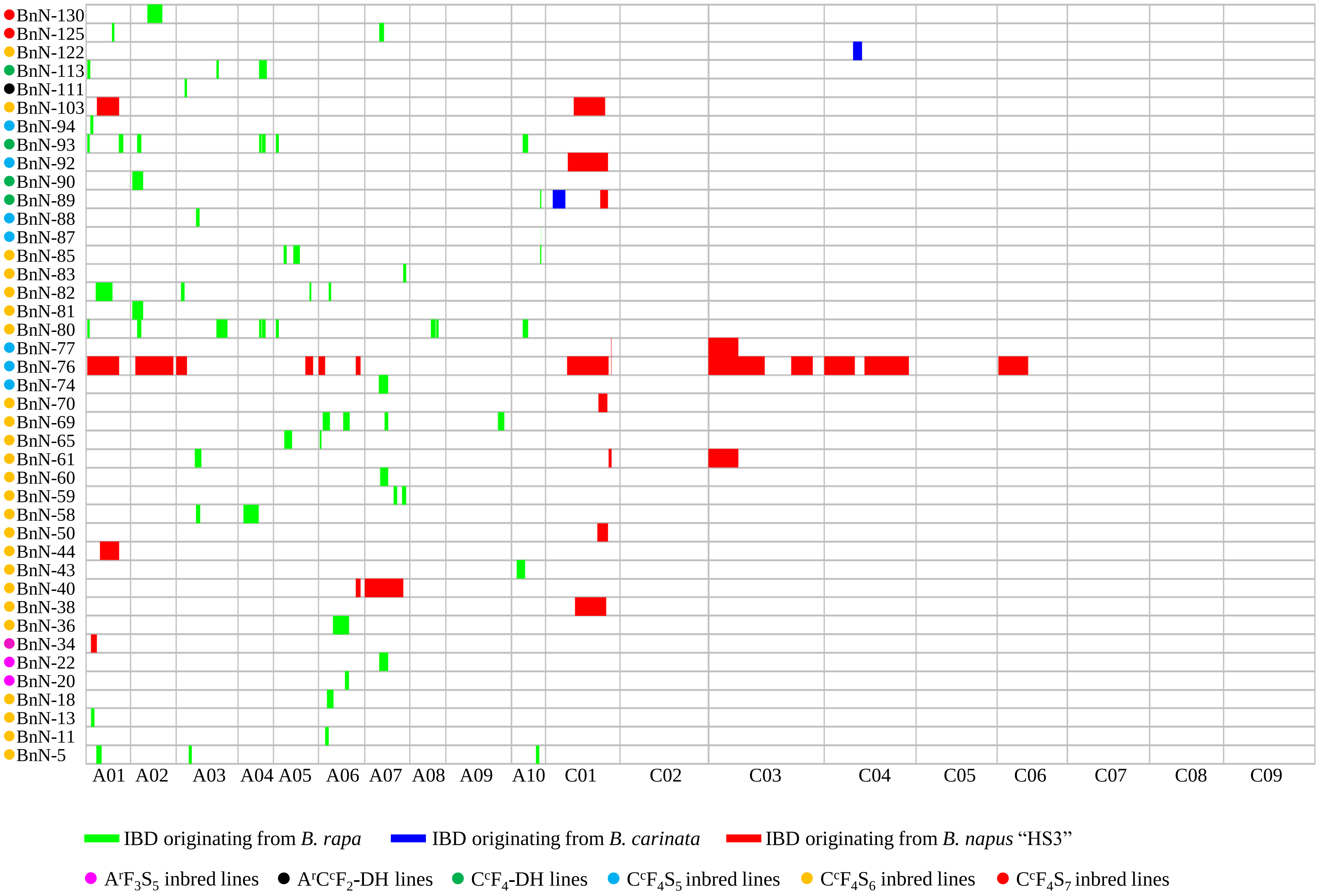
BnN-130
BnN-125
BnN-122
BnN-113
BnN-111
BnN-103
BnN-94
BnN-93
BnN-92
BnN-90
BnN-89
BnN-88
BnN-87
BnN-85
BnN-83
BnN-82
BnN-81
BnN-80
BnN-77
BnN-76
BnN-74
BnN-70
BnN-69
BnN-65
BnN-61
BnN-60
BnN-59
BnN-58
BnN-50
BnN-44
BnN-43
BnN-40
BnN-38
BnN-36
BnN-34
BnN-22
BnN-20
BnN-18
BnN-13
BnN-11
BnN-5
A01 A02 A03 A04 A05 A06 A07 A08 A09 A10 C01 C02 C03 C04 C05 C06 C07 C08 C09
IBD originating from *B. rapa*
IBD originating from *B. carinata*
IBD originating from *B. napus* “HS3”
$A^rF_3S_5$ inbred lines
$A^rC^cF_2$-DH lines
C^cF_4-DH lines
$C^cF_4S_5$ inbred lines
$C^cF_4S_6$ inbred lines
$C^cF_4S_7$ inbred lines
